# Peripheral and neural correlates of self-harm in children and adolescents: a scoping review

**DOI:** 10.1186/s12888-022-03724-6

**Published:** 2022-05-04

**Authors:** Victoria M. Sparrow-Downes, Sara Trincao-Batra, Paula Cloutier, Amanda R. Helleman, Mina Salamatmanesh, William Gardner, Anton Baksh, Rishi Kapur, Nicole Sheridan, Sinthuja Suntharalingam, Lisa Currie, Liam D. Carrie, Arthur Hamilton, Kathleen Pajer

**Affiliations:** 1 Department of Family Medicine Residency Program, Memorial University of Newfoundland, NL St. John’s, Canada; 2 Department of Pediatrics Residency Program, Memorial University of Newfoundland, NL St. John’s, Canada; 3 CHEO Research Institute, Ottawa, ON Canada; 4 Department of Psychiatry, University of Ottawa, ON Ottawa, Canada; 5 School of Epidemiology and Public Health, University of Ottawa, ON Ottawa, Canada; 6 Research Fellow, Harbourfront Health Group, Grand Falls, NB Canada; 7 PhD Program, Department of Cognitive Science, Carleton University, Ottawa, ON Canada

**Keywords:** Suicide, Suicide attempt, Non-suicidal self-injury, Peripheral correlates, Neural correlates, Children, Adolescents

## Abstract

**Background:**

Self-harm in children and adolescents is difficult to treat. Peripheral and neural correlates of self-harm could lead to biomarkers to guide precision care. We therefore conducted a scoping review of research on peripheral and neural correlates of self-harm in this age group.

**Methods:**

PubMed and Embase databases were searched from January 1980-May 2020, seeking English language peer-reviewed studies about peripheral and neural correlates of self-harm, defined as completed suicide, suicide attempts, suicidal ideation, or non-suicidal self-injury (NSSI) in subjects, birth to 19 years of age. Studies were excluded if only investigating self-harm in persons with intellectual or developmental disability syndromes. A blinded multi-stage assessment process by pairs of co-authors selected final studies for review. Risk of bias estimates were done on final studies.

**Results:**

We screened 5537 unduplicated abstracts, leading to the identification of 79 eligible studies in 76 papers. Of these, 48 investigated peripheral correlates and 31 examined neural correlates. Suicidality was the focus in 2/3 of the studies, with NSSI and any type of self-harm (subjects recruited with suicidality, NSSI, or both) investigated in the remaining studies. All studies used observational designs (primarily case-control), most used convenience samples of adolescent patients which were predominately female and half of which were recruited based on a disorder. Over a quarter of the specific correlates were investigated with only one study. Inter-study agreement on findings from specific correlates with more than one study was often low. Estimates of Good for risk of bias were assigned to 37% of the studies and the majority were rated as Fair.

**Conclusions:**

Research on peripheral and neural correlates of self-harm is not sufficiently mature to identify potential biomarkers. Conflicting findings were reported for many of the correlates studied. Methodological problems may have produced biased findings and results are mainly generalizable to patients and girls. We provide recommendations to improve future peripheral and neural correlate research in children and adolescents, ages 3-19 years, with self-harm.

**Supplementary Information:**

The online version contains supplementary material available at 10.1186/s12888-022-03724-6.

## Background

Self-harm, defined as completed suicide, attempted suicide, suicidal ideation, or non-suicidal self-injury (NSSI), is a significant health problem in children and adolescents worldwide [1, 2]. The third leading cause of death in 10–19 year-olds in England is suicide [3] and suicide rates in 15–19-year-old British and Welsh girls increased by 13.2% from 2010 to 2017 and in boys by 5.9% [4]. Completed suicide was the second leading cause of death in the US in 2019 for 10–19 year-olds (https://webappa.cdc.gov/sasweb/ncipc/leadcause.html). Studies from several countries also show that completed suicides, suicide attempts, or suicidal ideation in children younger than 12 are no longer unusual [5–9].

Completed suicide is associated with previous suicide attempts, suicidal ideation [10, 11], and NSSI [12, 13]. The issue of NSSI is important when discussing children and adolescents, as it has been reported to occur in up to 35% of the adolescent population worldwide [14, 15] and in nearly 8% of the general population of 7–8 year-olds [16]. Moreover, in a large clinical sample of 3–6 year-olds with major depressive disorder (MDD), rates of NSSI, suicidal ideation, and suicide attempts were present in 21.3, 19.1 and 3.5% of the children, respectively [17]. Recent data demonstrate that the younger the age of onset, specifically if younger than 13 years of age, the more severe and protracted the course of NSSI and suicidality [18].

In addition to completed suicide, children and adolescents with self-harm have poorer adult outcomes with lower educational and occupational attainment and more mental and physical health problems [19, 20]. Ideally, successful treatment would not only reduce the risk of completed suicide or other forms of self-harm, but also facilitate healthy development.

Unfortunately, we do not yet have an evidence-based, validated set of clinical treatment guidelines for treating pediatric self-harm [21]. Despite a growing number of encouraging treatment studies in recent years, systematic reviews and meta-analyses conclude that replication of positive effects from dialectical behaviour therapy (DBT) is needed and that more clinical trial research is still required for mentalization and family-based therapies to determine efficacy [22–24] Moreover, gaps in this corpus of work include an absence of studies about treatments for self-harm in children younger than 12 years and a paucity of trials for particularly vulnerable sub-groups such as those in care [25] and non-cis-gender children or adolescents [24]. No randomized controlled trials (RCTs) of pharmacological interventions for pediatric self-harm have been published and the results from non-RCT studies using adaptations of adult medication treatments have been disappointing [26–28].

Developing better treatments likely requires more detailed characterization of this complex population. Clinical biomarkers have the potential to advance such characterization. The first stage in biomarker development is the identification of valid and reliable peripheral, neural, or genetic correlates of clinical symptoms, treatment response, or long-term outcomes [29].

Three recent reviews have summarized the literature on genetic correlates of self-harm, including in children or adolescents [30–32]. Therefore, we focused our study on research covering peripheral and neural correlates. Eight reviews summarized research that included children and adolescents from birth to 19 years of age, six of which were done before 2016 [33–40]. A total of 31 studies (15 of peripheral correlates and 16 of neural correlates) were presented, but all these reviews except for one [34], combined findings on children and adolescents with the data from adults. An additional review published after we had submitted our manuscript also combined pediatric and adult data [41]. Of the nine publications, only one was a systematic review [34], although most of them took a systematic approach to finding studies.

Not differentiating child and adolescent data from those obtained from young adults in their twenties may be misleading in developing biomarkers for these younger age groups. Neuroimaging research shows that brain development continues into the late twenties, but the risk factors and clinical profiles of self-harming children and adolescents differ from those reported in young adults in their twenties [18, 42, 43]. For example, compared to adults who attempted suicide, adolescents had a significantly higher number of previous attempts, were more likely to be responding to interpersonal problems, and were more likely to use medication for self-poisoning [44]. Several reviews indicate that imaging findings may be different in self-harming adolescents than those reported for adults, e.g., in decision-making and impulsivity [38]. Moreover, adolescent hopelessness, loneliness, or impulsivity are less strongly related to self-harm than they are in adults [35]. Adolescents’ social risk factors are also different: parent-child conflict, school stressors, vicissitudes of early romantic relationships, victimization from bullying, and internet addiction and stress [37, 45]. These factors may affect associations between self-harm and biological correlates differently in children and adolescents than in young adults.

The discovery of clinical peripheral and neural biomarkers requires evidence of reliable and valid biological correlates studied in samples from the targeted population, which in this case is children and adolescents younger than 19 years of age. Identifying potential diagnostic, treatment, or prognostic biomarkers often begins with a quantitative synthesis of such a body of research. To our knowledge, such a synthesis does not exist for peripheral and neural correlates of all types of self-harm in children and adolescents. Thus, in preparation for a systematic review and meta-analysis, we conducted a scoping review of this corpus of work.

## Methods

Scoping reviews are designed to 1) identify and characterize studies in a body of research; 2) summarize how the research is conducted; 3) identify factors that can affect findings; 4) delineate research gaps; and 5) present implications for researchers (instead of for clinicians, as with systematic reviews) [46]. We used the Joanna Briggs Institute (JBI) structure for scoping reviews [47] and our review followed the Preferred Reporting Items for Systematic Reviews and Meta-Analyses-Scoping Reviews (PRISMA-ScR) guidelines, as summarized in the PRISMA-ScR checklist [48] (Supplement 1).

### Search strategy and information sources

Our search strategy used terms mapping onto constructs of age, e.g. ‘child’, ‘youth’, ‘adolescent’; self-harm, e.g. ‘suicide’, ‘self-injury’, ‘suicidality’, ‘non-suicidal self-injury’; and the broad search term of ‘biological correlates’, in addition to specific correlates obtained from previous reviews categories of correlates, e.g., ‘nutrition’ and ‘neurotransmitters’. Medical Subject Headings (MeSH terms) and keywords were incorporated into the search strategy. The searches were conducted in the PubMed and EMBASE databases from 1980 onwards. Searching was initiated on June 17, 2018, updated on September 13, 2019, and again on May 6, 2020. No studies published after May 6, 2020, were reviewed. Reference lists from studies obtained or reviews were also examined for missed studies. Any discovered were processed in the same manner as those found with the searches. Gray literature was not searched. Details of the search strategy are in Supplement 2.

### Eligibility criteria

Eligibility criteria were established a priori. A study was included if the following were investigated: 1) suicidality, defined as completed suicide, attempted suicide, suicide plans, or suicidal ideation; 2) NSSI, defined as self-harm of any type, e.g., cutting or burning, without intention to kill oneself; 3) any type of self-harm, i.e., not designated as strictly suicidality or NSSI; 4) participants with ages birth − 19 years; and 5) peripheral or neural biological correlates, i.e., objective, biological peripheral or neural data collected with non-invasive methods. Studies with participants older than 19 years were included if data were reported separately on those within our age range. Only peer-reviewed studies written in English were included. There were no restrictions on study location nor were there restrictions on how study subjects’ self-harm was defined. Every study was included if it focused on self-harm, whether participants were classified by diagnostic criteria from an edition of the Diagnostic and Statistical Manual (DSM), a version of the International Classification of Diseases (ICD), questionnaires, scales, interviews, or clinical records.

A study was excluded if it: 1) examined genetic correlates; 2) only investigated self-harm in patients as a function of severe intellectual or developmental disability syndromes, e.g., Lesh-Nyhan syndrome or tuberous sclerosis complex; or 3) was a conference abstract, review or case report.

### Screening process and data extraction

Covidence was used to assist in the processing of records [49]. Duplicates were removed from the abstracts, followed by a three-step process of blinded assessments by pairs of co-authors. Titles and abstracts were screened against inclusion and exclusion criteria, and those still eligible were then subjected to full-text screening. The remaining eligible studies were then subjected to full-text information extraction, using a spreadsheet adapted from the Strengthening the Reporting of Observational Studies in Epidemiology (STROBE) criteria [50] Disagreements between raters were resolved through consensus.

### Assessment of risk of bias

Although not required in a scoping review, to better characterize the research and formally assess factors that could affect findings, we estimated the risk of bias in each study. We used one of three tools, depending on study design: the *Quality Assessment Tool for Case-Control Studies,* the *Quality Assessment Tool for Observational Cohort and Cross-Sectional Studies,* or the *Quality Assessment Tool for Pre-Post Intervention Studies* from the U.S. National Heart, Lung, and Blood Institute (https://www.nhlbi.nih.gov/health-topics/study-quality-assessment-tools). These rating tools evaluate study methods and implementation as sources of bias, including sample characteristics (subject selection, participation, attrition), confounding, study power, and estimation of causality between exposures or interventions and outcomes. This system does not generate a score per study, but the items inform a rater’s qualitative assignment of a rank; Poor (high risk of bias), Fair (medium risk of bias), or Good (low risk of bias). Ratings were done by four raters in pairs, blind to each other’s work. Disagreements were resolved by the senior author who reviewed each study in question, blind to ratings from the others.

## Results

**Fig. 1:**
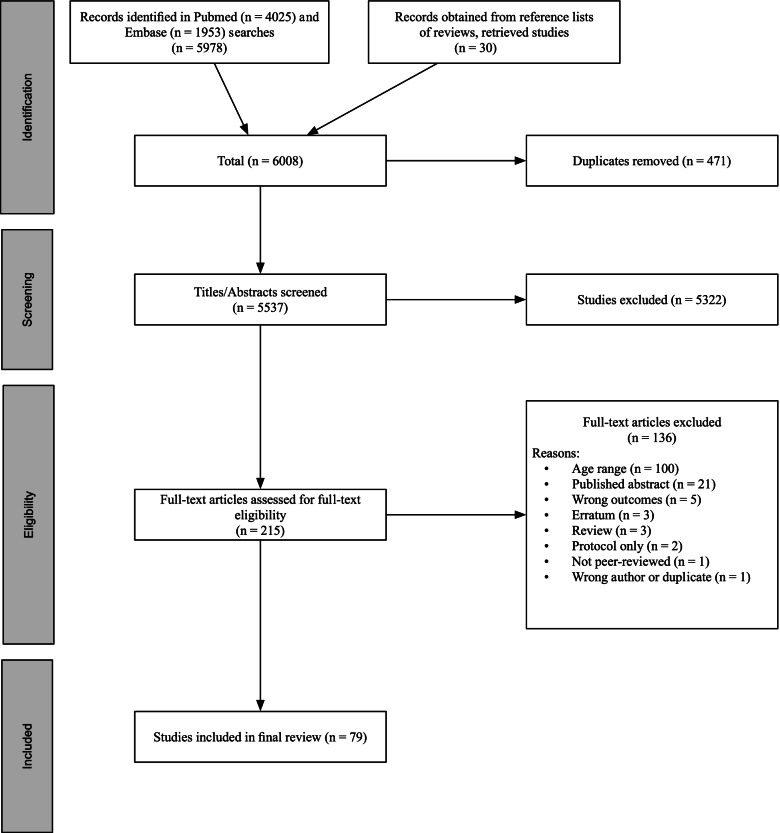
PRISMA Diagram

Figure 1 displays the PRISMA diagram of article processing. We located 4025 abstracts in PubMed and 1953 in Embase, resulting in a total of 5978 records. Back-searching reference lists identified 30 more records meeting eligibility criteria, for a total of 6008 articles processed. Removal of 471 duplicates left 5537 records for title/abstract screening, which found that 5322 articles were ineligible for inclusion. The full-text screening was conducted with the remaining 215 studies, and 136 were excluded for the reasons listed in Fig. 1. The most common reasons for exclusion were that studies were out of the age range or did not study a biological correlate.

The final number of studies retained was 79, including three papers that collected data on two different specific correlates (see Table 1) [51–53]. Therefore, for any results concerning the total number of studies, we use 79, as this refers to the number of studies of specific correlates. However, since these three papers conducted their research on the same sample for both specific correlates, we only used each paper once when summarizing designs, sample characteristics, and risk of bias.

**Table 1:** Studies reviewed, type of self-harm, designs, specific correlates studied

Citation, Country	Type of Self-harm	Study Design	Specific Correlate
***Peripheral Correlates***			
Robbins, Alessi 1985 US [54]	Suicidality	Case-control	Circadian rhythm, hypothalamic pituitary adreanal (HPA) axis, cortisol
Rosenthal et al. 1986 US [55]	Suicidality	Case-control	Circadian rhythm, HPA axis, cortisol
Dahl et al. 1991a US [56]	Suicidality	Case-control	Circadian rhythm, HPA axis, cortisol
Dahl et al. 1992a US [57]	Suicidality	Case-control	Circadian rhythm, HPA axis, cortisol
Ghaziuddin et al. 2014 US [58]	Suicidality	Case-control	Reactivity, HPA axis, cortisol
Young et al. 2010 Scotland [59]	Suicidality	Cross-sectional	Circadian rhythm, HPA axis, cortisol
Pfeffer et al. 1991 US [60]	Suicidality	Cohort	Circadian rhythm, HPA axis, cortisol
Giletta, et al. 2015 US [61]	Suicidality	Cohort	Reactivity, HPA axis, cortisol, predicting symptoms
Eisenlohr-Moul et al. 2018 US [62]	Suicidality	Cohort	Reactivity, HPA axis, cortisol, predicting symptoms
Reichl et al., 2016 Germany [63]	NSSI	Case-control	Circadian rhythm, HPA axis, cortisol
Klimes-Dougan et al. 2019 U S[64]	NSSI	Case-control	Reactivity, HPA axis, cortisol
Reichl, et al. 2019 Germany [65]	NSSI	Case-control	Circadian rhythm, Reactivity, HPA axis, cortisol
Beauchaine et al. 2015 US [66]	Any self-harm	Case-control	Circadian rhythm, HPA axis, cortisol
Plener et al. 2016 Germany [67]	Any self-harm	Cohort	Reactivity, HPA axis, cortisol
Yang et al., 2019 Hungary [68]	Suicidality	Case-control	Reactivity autonomic nervous system (ANS), cardiovascular (CV) system
Giletta et al., 2017 US [69]	Suicidality	Cohort	Reactivity, ANS, CV system, prediction symptoms
Koenig et al. 2017a Germany [70]	NSSI	Case-control	Resting, ANS, CV system
Crowell et al. 2005 US [51]	Any self-harm	Case-control	Resting & reactivity, ANS, CV system, skin conductance
Crowell et al. 2012 US [71]	Any self-harm	Case-control	Resting & reactivity, ANS, skin conductance
Wielgus et al. 2016 US [72]	Any self-harm	Cohort	Resting & reactivity, ANS, CV system, prediction symptoms
Aldrich et al. 2018 US [73]	Any self-harm	Cohort	Reactivity, ANS, skin conductance, prediction symptoms
Kaess et al. 2012 Germany [74]	NSSI	Case-control	Reactivity, HPA axis & ANS, cortisol, CV system
Koenig et al. 2017b Germany [75]	NSSI	Case-control	Reactivity, HPA axis & ANS, cortisol, CV system
Modai et al. 1989 Israel [76]	Suicidality	Case-control	Platelet serotonin function
Ambrosini et al. 1992 US [77]	Suicidality	Case-control	Platelet serotonin function
Pfeffer et al. 1998 US [78]	Suicidality	Case-control	Serotonin, precursor levels, platelet function
Tyano et al. 2006 Israel [79]	Suicidality	Case-control	Serotonin levels
Pine et al. 1995 US [80]	Suicidality	Cohort	Platelet serotonin function
Clark et al. 2003 US [81]	Suicidality	Cohort	Precursor levels, prediction symptoms
Crowell et al. 2005 US [51]	Any self-harm	Case-control	Serotonin levels
Crowell et al. 2008 US [82]	Any self-harm	Case-control	Serotonin levels
Dahl et al., 1990 US [83]	Suicidality	Case-control	Sleep characteristics
Dahl et al. 1991b US [84]	Suicidality	Case-control	Sleep characteristics
Emslie et al. 1994 US [85]	Suicidality	Case-control	Sleep characteristics
McCracken et al. 1997 US [86]	Suicidality	Case-control	Sleep characteristics
Boafo et al. 2019 Canada [87]	Suicidality	Case-control	Sleep characteristics
Singareddy et al. 2013 US [88]	Suicidality	Cohort	Sleep characteristics
Bilgiç et al. 2020 Turkey [89]	Suicidality	Case-control	Neurotrophin levels
Falcone et al. 2010 US [52]	Suicidality	Case-control	S100 calcium-binding protein B (S100B) levels
Falcone et al. 2015 US [90]	Suicidality	Case-control	S100B levels
Kavurma et al. 2017 Turkey [91]	Any self-harm	Case-control	Neurotrophin levels
Gabbay et al. 2009 US [92]	Suicidality	Case-control	Cytokine levels
Falcone et al. 2010 US *(supplement data )*[52]	Suicidality	Case-control	Cytokine levels
Amitai et al. 2020 Israel [93]	Suicidality	Non-controlled pre-post intervention	Cytokine levels, predicting side effects
Glueck et al. 1994 US [94]	Suicidality	Case-control	Lipid levels
Plana et al. 2010 Spain [95]	Suicidality	Case-control	Lipid levels
Ryan et al. 1988 US [96]	Suicidality	Case-control	Reactivity, growth hormone
Dahl et al.1992b US [97]	Suicidality	Case-control	Circadian rhythm, growth hormone
***Neural Correlates***			
Pan et al. 2011 US [98]	Suicidality	Case-control	Brain activity, response inhibition
Pan et al. 2013a US [53]	Suicidality	Case-control	Brain activity, facial emotion processing
Pan et al. 2013b US [99]	Suicidality	Case-control	Brain activity, decision-making
Quevedo et al. 2016a US [100]	Suicidality	Case-control	Brain activity, emotion self-identity
Harms et al. 2019 US [101]	Suicidality	Case-control	Brain activity, social interaction
Oppenheimer et al. 2020 US [102]	Suicidality	Case-control	Brain activity, social interaction
Plener et al. 2012 Germany [103]	NSSI	Case-control	Brain activity, emotion processing
Groschwitz et al. 2016 Germany [104]	NSSI	Case-control	Brain activity, social interaction
Quevdo et al. 2016b US [105]	NSSI	Case-control	Brain activity, emotion self-identity
Brown et al. 2017 Germany [106]	NSSI	Case-control	Brain activity, social interaction
Perini et al. 2019 Sweden [107]	NSSI	Case-control	Brain activity, social interaction
Poon et al. 2019 US [108]	NSSI	Cross-sectional	Brain activity, reward processing
Sauder et al., 2016 US [109]	Any self-harm	Case-control	Brain activity, reward processing
Pan et al. 2013a US [53]	Suicidality	Case-control	Functional connectivity, facial emotion processing
Alarcon et al. 2019 US [110]	Suicidality	Case-control	Functional connectivity, emotion self-identity
Ordaz et al. 2018 US [111]	Suicidality	Cross-sectional	Functional connectivity, resting state
Schreiner al. 2018 US [112]	Suicidality	Cross-sectional	Functional connectivity, resting state
Schwartz et al. 2019 US [113]	Suicidality	Cohort	Functional connectivity, resting state, predicting symptom change
Santamarina-Perez et al. 2019 US [114]	NSSI	Cohort	Functional connectivity, resting state, predicting treatment effect
Tavakoli et al. 2018 Canada [115]	Suicidality	Case-control	Event-related potential, attention capture
Tsypes, et al. 2019 US [116]	Suicidality	Case-control	Event-related potential, reward-loss
Pegg et al. 2020 US [117]	Suicidality	Case-control	Event-related potential, reward-loss
Tsypes et al. 2018 US [118]	NSSI	Case-control	Event-related potential, reward-loss
Graae et al. 1996 US [119]	Suicidality	Case-control	Resting state, brain wave asymmetry
Lewis et al. 2019 US [120]	Suicidality	Non-controlled pre-post intervention	Reactive intracortical inhibition, post-treatment
Ho et al. 2018 US [121]	Suicidality	Cohort	Gray matter volume, predicting symptoms
Ando et al. 2018 Germany [122]	NSSI	Case-control	Gray matter volume
Beauchaine, et al. 2019 US [123]	Any self-harm	Case-control	Gray matter volume
Pan et al., 2015 US [124]	Suicidality	Case-control	Gray, white matter volumes
Goodman et al. 2011 US [125]	Any self-harm	Case-control	Gray and white matter volumes
Jovev et al. 2008 US [126]	Any self-harm	Cross-sectional	Pituitary gland volume

 Self-harm focus in the studies was defined in one of three ways with three types of samples: suicidality (subjects only with suicide attempts, plans, or ideation), NSSI (subjects only with self-harm without intention to die), or what we labeled ‘any self-harm’ (subjects who manifested suicidality, NSSI, or both, but the samples were mixed with regards to types of self-harm). Most studies, 65% (51/79), focused on subjects with suicidality, while 19% (15/79) studied NSSI, and 16% (13/79) investigated participants recruited with Any Self-Harm.

**Fig. 2:**
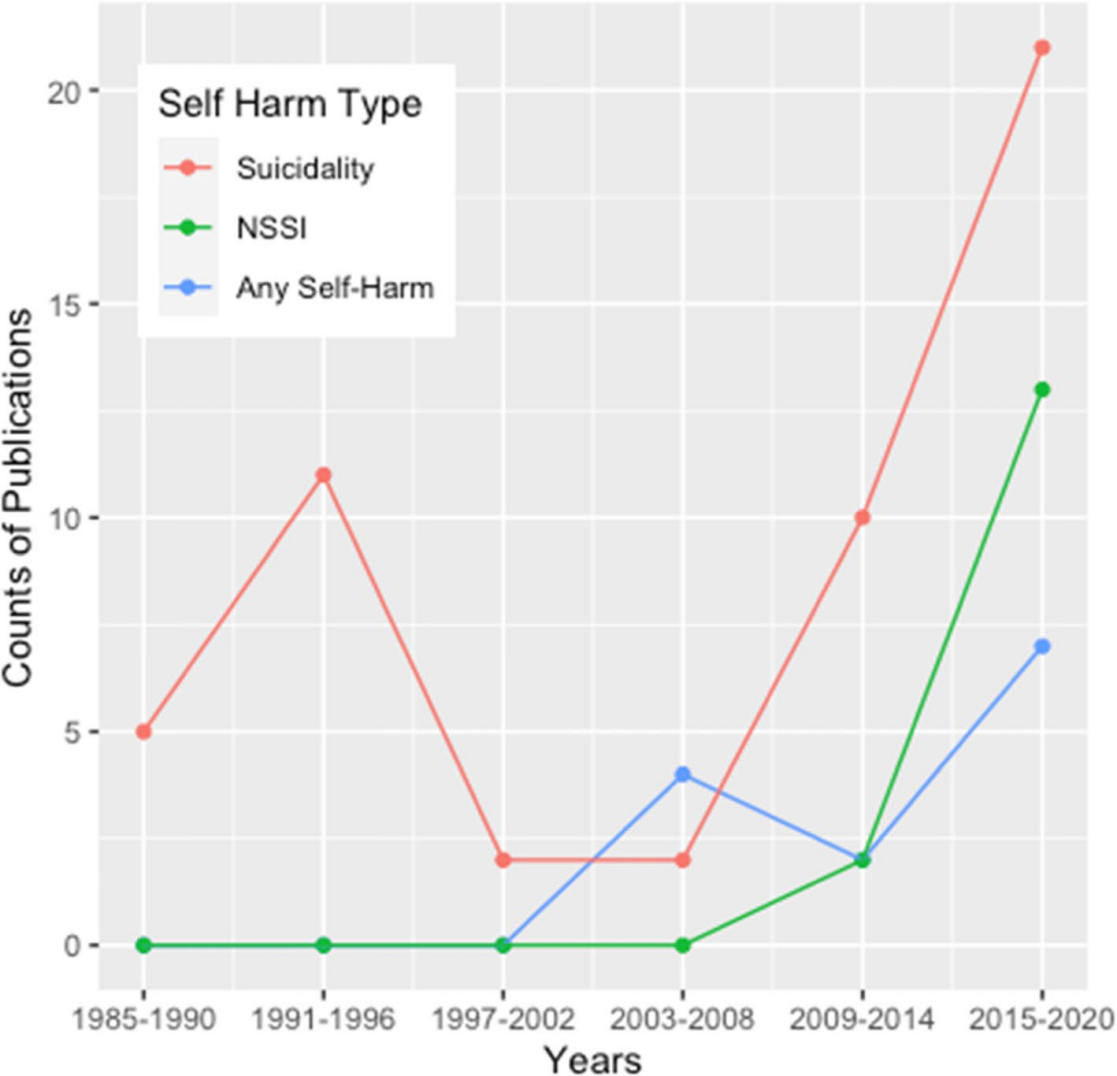
Publication dates by type of self-harm studied

Publication dates ranged from January 1985 to May 2020. There was a relationship between time of publication and type of self-harm studied, as shown in Fig. 2. The counts of papers on suicidality have more than doubled every 5 years since 2009. Research on studies of any self-harm first showed up in 2005, whereas NSSI papers did not appear until 2012. Numbers of studies about any type of self-harm have increased in the past 5 years, as have NSSI studies in the past 10 years. However, work on suicidality has continued to show the largest growth.

**Fig. 3:**
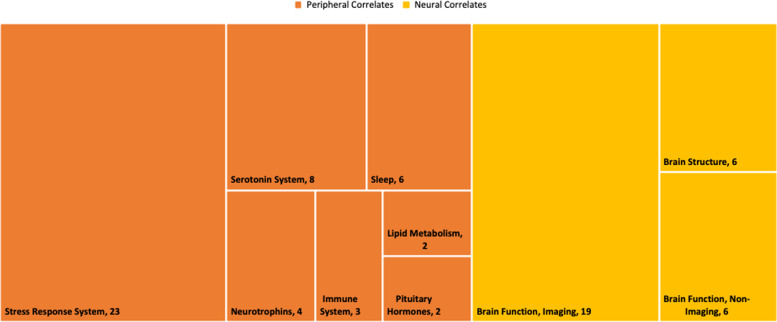
Tree map of peripheral and neural correlate sub-categories and number of studies

We found 48 studies of peripheral correlates and 31 of neural correlates. We categorized these into seven sub-types of peripheral correlates and three sub-types of neural correlates (see Fig. 3). The most frequently-studied sub-category in the peripheral correlates was the stress response system and for the neural correlates, it was brain function with imaging. There were no replication studies.

Most of the studies were conducted in the US. Germany was the location for eight studies. Three or fewer were done in Israel, Canada, Turkey, Scotland, Spain, Sweden, and Hungary.

### Study designs, samples, identifying self-harm, specific correlates studied bias/quality ratings

Table 1 displays the studies included organized by whether they were in the peripheral or neural category, the type of self-harm that was the focus of the study, the design, and the specific correlate studied [51–126]. Details about samples, methods, and findings, as well as risk of bias rating for each study can be found in Supplement 3 (peripheral correlates) or 4 (neural correlates).

#### Designs

Proportions of study designs were similar across correlate categories. Case-control designs were most frequently used, in 74% (34/46) of the peripheral correlates studies and 70% (21/30) of the neural correlate studies. Cross-sectional or cohort designs were employed in similar proportions across correlate categories (24% or 11/46, peripheral and 23% or 7/30 of the neural correlate studies). Likewise, each category had one non-controlled pre-post intervention study.

#### Samples

Table 2 and Supplements 3 and 4 show that sample characteristics varied widely across studies. Samples in the peripheral correlates more frequently comprised subjects with suicidality: 70% (32/46), compared to 60% of the neural correlate papers (18/30). The neural correlate data more often came from samples of participants with NSSI 30% (9/30) compared to only 13% (6/46) of the studies of peripheral correlates. The proportions of papers in each category studying participants with any type of self-harm were more similar, 17% (8/46) in the peripheral correlates and 13% (4/30) in the neural correlates research.

**Table 2:** Sample characteristics

Characteristic	Peripheral Correlates n (%) N of studies = 46^1^	Neural Correlates n (%) N of studies = 30^1^
***Self-Harm Type***		
Suicidality	32 (70%)	17 (57%)
NSSI	6 (13%)	9 (30%)
Any Self-Harm	8 (17%)	4 (13%)
***Sample Size***		
9-20	2 (4%)	3 (10%)
21-50	13 (28%)	15 (50%)
51-100	13 (28%)	7 (23%)
101-200	12 (26%)	5 (17%)
>200	6 (13%)	0 (0%)
***Age Group***		
Children (3-11 years old)	2 (4%)	2 (7%)
Adolescents (12-19 years old)	30 (65%)	20 (67%)
Both	14 (30%)	8 (27%)
***Source of Subjects***		
Clinical setting	9 (20%)	4 (13%)
Community	5 (11%)	4 (13%)
Clinical setting & community	29 (63%)	15 (50%)
Previous research, registry	3 (7%)	6 (20%)
Not given	0 (0%)	1 (3%)
***Recruitment Diagnosis***		
Depression	18 (39%)	12 (40%)
Other	6 (13%)	3 (10%)
None	22 (48%)	15 (50%)
***Types of Controls***^2^		
Healthy controls	21 (62%)	13 (57%)
Psychiatric controls	7 (21%)	1 (4%)
Both	6 (18%)	9 (39%)
***≥85% Girls***		
Yes	14 (30%)	8 (27%)
No	31 (67%)	21 (70%)
Not given	1 (2%)	1 (3%)

^1^Total number of studies adjusted for 2 studies in peripheral correlates and 1 study in neural correlates that used the same samples for two specific correlates

^2^ Denominators are studies using controls: n = 34, peripheral correlates; *n* = 23, neural correlates

Sample sizes for the entire body of work ranged from 9 to 1268. The range for the peripheral correlates’ samples was 9–1258 subjects (median = 62.5), but the neural correlates’ range was 10–152 participants (median = 44). Adolescents were studied most often, with only two studies in each category exclusively examining children younger than 12. Nearly the same percentage of studies in each correlate category used combined samples of children and adolescents.

Many studies (63% (29/46) of the peripheral correlate studies and 50% (15/30) of the neural correlates) recruited convenience samples of clinical cases from inpatient units or outpatient clinics. A number of these studies also recruited from the community, but this process was always to obtain healthy controls, not self-harming children or adolescents who were not patients. Self-harming participants in cross-sectional and cohort studies were also often collected as convenience samples, but several studies used samples of the general population recruited with population sampling methods.

Half of the studies in both correlate categories recruited subjects based on a clinical diagnosis, almost always major depressive disorder (MDD). Other disorders targeted subjects with borderline personality disorder, anxiety disorders, or psychosis mixed with all types of mood disorders. Several studies also recruited subjects with “MH concerns,” but no diagnosis was associated with this.

Of the studies that used control groups (34 peripheral and 23 neural studies), healthy control groups were the most common: 62% (21/34) for peripheral correlates and 57% (13/23) for neural correlates. Few studies used only psychiatric controls, but 39% (9/23) of neural correlate projects recruited healthy and psychiatric controls, and 18% (6/34) of the peripheral correlates used both.

Girls were studied more often than boys. The percentage of girls, averaged across all studies, was 72% and the range was 11 to 100%. Nearly a third of the studies investigated chiefly or entirely female samples, which we defined as > 85% girls: 30% (14/46) and 27% (8/30) in the peripheral and neural categories, respectively.

#### Methods to identify self-harm

Data classifying the type of self-harm were collected with six approaches: 1) self- or parent-report, 2) diagnostic interview, 3) clinician-rated scale/non-diagnostic interview, 4) combinations of the previous three approaches, 5) clinical records, or 6) non-standardized instruments created for the specific study. Data collection methods were similar in the peripheral and neural correlates research, but there was little inter-study consistency, as shown in Supplements 3 and 4.

The lack of consistency is illustrated by the finding that numerous different instruments were used within each of the first four approaches. Self-report data about self-harm were collected from one or more of 16 instruments, diagnostic interview data from one of five instruments, and information from clinician-rated scales/non-diagnostic interviews could have come from one or more of 14 instruments. Many of the instruments used were designed for adults and lacked psychometric data for use in the pediatric age group. Moreover, several of the instruments in all categories were not designed to assess self-harm and classified subjects based on answers to just a few questions (sometimes only one), e.g., the Youth Self-Report (YSR) [127] or older versions of the Kiddie-Schedule for Affective Disorders and Schizophrenia (K-SADS) [128].

The most frequently used approach was clinician rating scales/non-diagnostic interviews. Studies of peripheral correlates most often used the Self-Injurious Thoughts and Behaviors Interview (SITBI) [129], while the neural correlates research most frequently used the Columbia Suicide Severity Rating Scale (C-SSRS) [130]. The next most common data collection method was diagnostic instruments, usually with a version of the K-SADS. Self-report studies were the third most common, with the neural correlate studies using this strategy nearly twice as often as peripheral correlates.

Neural correlate research used the Suicidal Ideation Questionnaire (SIQ) [131] most frequently, in contrast to the YSR in peripheral correlate studies. Over a third of the neural correlate studies used a combination of methods, compared to only 15% (7/48) of the peripheral. Clinical records used to categorize subjects on self-harm were used in 10% (5/48) of the peripheral correlate work, although only 3% (1/31) of the neural correlate studies did this. Only one study used a non-standardized instrument. An unusual study used response latency to timed judgments of pairs of death-related and self-related words to categorize participants. Shorter latency to respond to death/me words than to life/me was categorized as implicit self-harm, differentiated from explicit self-harm defined by standard self-report measures. The authors suggested that this strategy may yield more reliable classification than self-report, especially in younger children who may have trouble articulating their feelings [121].

#### Specific correlates

Table 3 presents the 28 specific correlates measured in these studies and the number of methods and outcomes for each (see Supplements 3 and 4 for further details). Over a quarter (29% (8/28)) of the specific correlates were investigated in only one study. The remainder were examined with two to eleven studies, but even in clusters of studies about one specific correlate, there was heterogeneity in the methods used to measure the correlate. Research on event-related potentials (ERPs) in reward processing had the most consistent methods. In contrast, five methods were used to investigate the reactive function of the autonomic nervous system (ANS). Outcomes of interest also varied considerably, with studies of some specific correlates all focusing on the same outcome, e.g., reactivity of the HPA axis measuring changes in cortisol levels, while groups of other studies examined diverse outcomes, e.g., neural functional connectivity studies investigated six different outcomes.

**Table 3:** Specific correlates: number of studies, measurement methods, and outcomes

Specific Correlates, Number of Studies	Number of Methods	Number of Outcomes
***Stress Response System***		
Cortisol levels, circadian (*n* = 11)	3	1
Cortisol levels, reactive (*n* = 8)	4	1
ANS, resting (*n* = 2)	1	5
ANS, reactive (*n* = 8)	4	5
***Serotonin System***		
Platelet serotonin uptake or imipramine binding (*n* = 3)	2	2
Blood levels serotonin (*n* = 4)	2	1
Blood levels serotonin precursors (*n* = 1)	2	1
***Sleep***		
Total duration, stage duration, latency to onset, latency to stages, REM density (*n* = 6)	1	2
***Neuromodulators***		
Serum levels S100B (*n* = 2)	1	1
Serum levels neurotrophins (*n* = 2)	1	1
***Immune System***		
Serum/plasma levels cytokines (*n* = 3)	1	2
***Lipid Levels***		
Serum cholesterol, triglycerides (*n* = 2)	1	2
***Pituitary Hormones***		
Circadian rhythm growth hormone (*n* = 1)	N/A	
Reactivity growth hormone (*n* = 1)	N/A	
***Brain Function, Imaging***		
Response inhibition (*n* = 1)	N/A	
Emotion processing (*n* = 3)	2	2
Decision-making (*n* = 1)	N/A	
Social interaction (*n* = 5)	3	1
Reward processing (*n* = 2)	2	1
Self-identity paradigm (*n* = 3)	2	2
Functional connectivity (*n* = 6)	1	3
***Brain Function, Non-Imaging***		
Event-related potentials, attention capture (*n* = 1)	N/A	
Event-related potentials, reward processing (*n* = 3)	1	3
Alpha waves, resting (*n* = 1)	N/A	
Intracortical inhibition (*n* =1)	N/A	
***Brain Structure***		
Gray matter volume (*n* = 3)	1	3
Gray and white matter volume (*n* = 2)	1	2
Pituitary gland volume (*n* = 1)	N/A	

#### Risk of bias ratings

Table 4 summarizes the risk of bias assessment rating results, organized by correlate sub-categories. An estimate of Good was given to 37% (29/79) studies; Fair to 57% (45/79), and Poor to 4% (4/79). Inter-rater agreement was moderate (*k* = 0.23) [132]. Neuromodulator and lipid metabolism had the highest percentage of Good studies with 100% in each. However, there were only four and two studies in each, respectively. And the two studies of S100B levels appeared to be overlapping samples, although this was not explicitly stated. Slightly over half of the twenty-three studies of the stress response system were rated as Good, and likewise half of the sleep studies. The remaining studies were judged to be Fair. The high percentage (50%) of studies rated as Poor in the pituitary hormones sub-category is due to only having two papers for this correlate and one of them rated as Poor.

**Table 4:** Summary of risk of bias ratings per correlate sub-category

	Number of studies with each rating (%)		
	**Good**	**Fair**	**Poor**
**Peripheral Correlates Sub-Categories (Total numbers of studies)**			
Stress Response System (*n* = 23)	12 (52%)	9 (39%)	2 (9%)
Serotonin System (*n* = 8)	3 (38%)	4 (50%)	1 (12%)
Sleep (*n* = 6)	3 (50%)	3 (50%)	0 (0%)
Neuromodulators (*n* = 4)	4 (100%)	0 (0%)	0 (0%)
Immune System (*n* = 3)	1 (33%)	2 (67%)	0 (0%)
Lipid Metabolism (*n* = 2)	2 (100%)	0 (0%)	0 (0%)
Pituitary Hormones (*n* = 2)	0 (0%)	1 (50%)	1 (50%)
**Neural Correlates Sub-Categories (Total number of studies)**			
Brain Function, Imaging Studies (*n* = 19)	3 (16%)	16 (84%)	0 (0%)
Brain Function, Non-Imaging Studies (*n* = 6)	1 (17%)	5 (83%)	0 (0%)
Brain Structure (*n* = 6)	1 (17%)	5 (83%)	0 (0%)

The most common reasons that studies were not scored as Good were: no sample size justification or power analysis, measurements of specific correlates were not blind to self-harm status (of particular importance in cohort studies), self-harm participants and control group members were not chosen at random from potential subjects (of particular importance in case-control studies), and analyses of the association between self-harm and specific correlates did not account for confounding variables, e.g. medication usage or gender. We did not find a relationship between ratings and publication date. As this was a scoping review, we did not exclude studies rated as Poor. Neither did we weight individual study findings by bias scores in the next section.

### Summary of study findings by type of self-harm

Table 5 presents a high-level summary of individual study findings, organized by the type of self-harm investigated. We could not calculate the number of unique participants studied for each correlate category because some studies appeared to have used the same sample in different studies, although that was rarely stated. At least one significant association with some type of self-harm was reported for all specific correlates, except three: neurotrophins [89, 91], neuroimaging and response inhibition [98] and neuroimaging and decision-making [99] (see Supplements 3 and 4). In the last two studies, subjects with MDD plus suicidality and healthy controls showed no differences in neuroimaging findings, but subjects with MDD did have aberrant responses.

**Table 5:** Overview of findings by type of self-harm

**Correlate Category**	**Type of Self-Harm Study**		
	**Suicidality **	**NSSI **	**Any Type of Self-Harm **
**Peripheral**	• *HPA Axis, Circadian rhythm, cortisol: *not associated (3/6). Associated with cortisol dysregulation (3/6). • *HPA Axis, Reactivity, cortisol*: associated with hyperreactivity (1/3), hyporeactivity (1/3), different pattern (1/3). • *ANS, Reactivity, cardiovascular measures*: Not associated (1/3). Decreased parasympathetic function (2/3), including symptom prediction. • *Serotonin System, platelets: *not associated imipramine binding sites (1/4). Not associated with serotonin uptake (1/4). Not associated with serotonin-induced aggregation (1/4). Associated with decreased imipramine binding sites (1/4). • *Serotonin System, serotonin levels:* Not associated with levels (1/2). Associated with higher levels (1/2). • *Serotonin System, precursor levels: *associated with lower tryptophan levels (1/2). 5-year suicidality associated with low ratio tryptophan to other amino acids, not baseline tryptophan levels (1/2). • *Sleep Characteristics*: not associated (2/6). Associated with longer sleep, shorter Stage 3, shorter delta sleep, more rapid eye movement (REM) after scopolamine (1/6). Associated with longer sleep latency (2/6), longer REM latency, higher percentage NREM1, higher REM density (1/6), higher percentage REM sleep (1/6). • *Neuromodulators*: Not associated with BDNF (2/2), or GDNF, NGF, NTF3 (1/2). Associated with increased levels of S100B protein (2/2). • *Immune System, cytokine levels:* associated with decreased TNFα, increased IFN- (1/3), increased IL-ß, IL-8 (1/3). Antidepressant suicidality associated with higher increase IL-6 (1/3). • *Lipid Metabolism:* not associated with triglyceride levels (1). Associated with lower cholesterol (2/2). • *Growth Hormone:* associated with blunted reactivity (1). Associated with dysregulated circadian secretion (1).	• *HPA Axis, Circadian rhythm, cortisol: *associated with cortisol dysregulation (2/2). • *HPA Axis, Reactivity, cortisol*: associated with hyporeactivity (2/4), hyperreactivity (1/4), different pattern (1/4). • *ANS, Resting, cardiovascular measures*: not associated (1).	• *HPA Axis, Circadian rhythm, cortisol: *associated with cortisol dysregulation (1). • *HPA Axis, Reactivity, cortisol*: associated with hyporeactivity (1). • *ANS, Resting, cardiovascular measures*: not associated with parasympathetic tone (1/2). Associated with lower parasympathetic tone (1/2). • *ANS, Reactivity, cardiovascular measures*: not associated with parasympathetic function (1). • *ANS, Resting, skin conductance:* not associated with abnormal sympathetic system arousal (1/2). Associated with lower sympathetic arousal (1/2). • *ANS, Reactivity, skin conductance*: reactivity not associated with prediction symptoms (1/2). Not associated with abnormal sympathetic system arousal (1/2). • *Serotonin System, serotonin levels:* associated with lower blood levels (2/2).
**Neural**	• *Activity, Response inhibition*: not associated (1). • *Activity, Emotion processing: *angry faces – increased activation in ACG, bilateral sensory cortices, left dlPFC, right MTG, happy faces – decreased activation visual, sensory cortices, PFC, ACG.(1) • *Activity, Decision-making*: not associated (1). • *Activity, Self-identity processing*: decreased activation in midline cortical, limbic structures for self-happy vs. other-happy faces (1) • *Activity, Social interaction*: associated in all scenarios with decreased activity insula, putamen, ACC, caudate, postcentral, precentral gyri (1). Associated with greater activation insula only if more peer victimization or daily negative experiences (1). • *Functional Connectivity, Emotion processing*: angry faces - decreased connectivity ACG to bilateral insulae (1). • *Functional Connectivity, Self-identity processing: *greater connectivity between amygdala, dlPFC, dmPFC, precuneus (1). • *Functional Connectivity, Intrinsic network coherence*: lower Executive Control Network coherence during resting state (1/2). Symptom improvement associated with increased coherence Salience Network (1/2). • *Functional Connectivity, Resting state*: associated with increased connectivity between left precuneus and primary motor, somatosensory cortices, MFG, SFG; decreased connectivity between left PCC, left cerebellum, left OC, temporal-occipital fusiform gyrus (1). • *Event-Related Potential, Attention capture*: associated with lower threshold involuntary attention switching (1). • *Event-Related Potential, Reward-loss: *associated with more activation to reward and loss (2/2). • *Brain Waves, Symmetry*: associated with left > right posterior alpha asymmetry (1). • *Cortical Inhibition, Post-treatment: *associated with increase cortical inhibition (1). • *Brain Structures, Gray matter volume:* prediction of symptoms associated with decreased volume of bilateral putamen, left caudate (1). • *Brain Structures, Gray and matter volume: *Not associated with white matter differences; associated reduced thickness in rSTG (1). • *Pituitary gland volume: *associated with increased volume (1).	• *Activity, Emotion processing*: associated with greater activation amygdala, hippocampus, bilateral ACC, but MDD explained findings (1). • *Activity, Social interaction: *associated with increased activation mPFC, vlPFC, parahippocampus (1/3). Associated with increase activation putamen (1/3). dmPFC, PCC, sgACC function during social anticipation predicted NSSI group (1/3). • *Activity, Self-identity processing: *Associated with greater activation limbic and cortical midline structures (1). • *Activity, Reward processing: *associated with greater activation bilateral putamen (1). • *Functional Connectivity, Resting state*: associated with reduced connectivity amygdala-mPFC network, predicted better response to treatment (1). • *Event-Related Potential, Reward-loss:* associated with more negative response to losses (1). • *Brain Structures, Gray matter volume: *associated with decreased volume ACC, insula (1).	• *Activity, Reward processing: *Associated with decreased activation putamen, OFC, bilateral amygdalae (1). • *Brain Structures*, *Gray matter volume:* associated with decreased volume bilateral insula cortices, rIFG (1). • *Brain Structures*, *Gray and matter volume:* associated with decreased gray and white matter volumes in BA24, higher white matter volume in BA23, but no difference in gray matter volume (1). • *Brain Structures, Pituitary gland:* associated with greater volume (1).

*ACC *Anterior Cingulate Cortex, *ACG *anterior cingulate gyrus, *BDNF *Brain-Derived Neurotrophin Factor, *BA23, BA 24 *Brodmann Area 23 and 24 (ventral posterior cingulate area), *dlPFC *Dorsolateral Prefrontal Cortex, *dmPFC *Dorsomedial Prefrontal Cortex, *GDNF *Glial-Derived Neurotrophin Factor, *GMV* Gray Matter Volume, *IFN-𝛾 *Interferon-Gamma, *IL-10 *Interleukin 10, *IL-1α *Interleukin 1-Alpha, *IL-1β *Interleukin 1-Beta, *IL-2 *Interleukin 2, *IL-4 *interleukin 4, *IL-6 *Interleukin 6, *IL-8 *Interleukin 8, *OC *Occipital Cortex, *mFC *medial frontal cortex, *mOFC *Medial Orbitofrontal Cortex, *mPFC *medial prefrontal cortex, *NGF *Nerve Growth Factor, *NTF3 *Neurotrophin-3 Factor, *OFC *Orbitofrontal Cortex, *PCC *Posterior Cingulate Cortex, *PF *Prefrontal, *rACC *Rostral Anterior Cingulate Cortex, *rdACG *Right Dorsal Anterior Cingulate Gyrus, *rIFG *Right Inferior Frontal Gyrus, *rMTG *Right Medial Temporal Gyrus, *rSTG *Right Superior Temporal Gyrus, *S100B *S100 - calcium-binding protein B, *sgACC *Subgenual Anterior Cingulate Cortex, *SN *Salience Network, *TNFα *Tumour Necrosing Factor-Alpha, *vLPFC *Ventrolateral Prefrontal Cortex, *WMV *White Matter Volume

Table 5 shows that, first, there are substantially more data from children and adolescents with suicidality than on subjects with NSSI or those from studies of any type of self-harm. Second, the findings for the specific correlates are inconsistent, even for the correlates with larger numbers of studies and even within a single type of self-harm. For example, studies of the association between suicidality and HPA axis reactivity reported hyperreactivity [61], hyporeactivity [62], and an aberrant secretion pattern [58] and in subjects with NSSI, three studies reported hyporeactivity [64, 67, 74] and one study reported hyperreactivity [75]. In an example from the neural correlates, brain activity in response to social interaction was associated with suicidality, but one study reported decreased activity in the insula [101] and the other reported increased activity [102]. Heterogeneity in the methods used to identify self-harm, sample characteristics, and measurement of the specific correlates or outcomes is so prevalent that it is difficult to interpret these discrepant findings.

A third feature of these findings evident in Table 5 is that there are stronger signals from some of the studies of specific correlates. These appear to be from groups of studies more methodologically similar and with lower risks of bias. For example, two studies with larger sample sizes, similar age ranges, outpatients and healthy controls, and similar proportions of girls investigated the association between neurotrophins and suicidality or any type of self-harm (for which data were analyzed separately). Correlate measurement and outcome methods were similar. Both were rated Good with regards to risk of bias. No association was found with any neurotrophin in either study. Similarly, two studies of lipid metabolism and suicidality both used samples of inpatients, clinical records to classify self-harm, used the same methods for measuring correlates, and compared the outcomes with normed levels in children and adolescents. Risk of bias ratings were Good for both studies. Both found that suicidality was associated with lower cholesterol.

Fourth, there are no clear patterns of findings, e.g., differences or similarities, for any of the specific correlates by self-harm sub-types. For most of the correlates, the issue is moot because there aren’t enough studies for comparison of findings by self-harm sub-type. But even with specific correlates having studies about each type of self-harm, e.g. the stress response system or neuroimaging responses to social interaction, it is difficult to determine if self-harm sub-type makes any difference in the results because of such wide inter-study methodologic variability.

Fifth, most studies examined correlates with designs and protocols that could contribute to development of diagnostic biomarkers. However, several are notable for correlate investigations that could inform advancement of prognostic [61, 62, 67, 69, 72, 73, 80, 81, 121] or treatment response biomarkers [113, 114, 120].

## Discussion

This scoping review contributes to research on peripheral and neural correlates of self-harm by summarizing data on children and adolescents ages 3 to 19 years, a demographic with social, developmental, and psychological characteristics of self-harm that can differ from those found in young adults [18, 35, 37, 38, 42–45]. Our work also advances knowledge on this topic by reviewing 79 studies in 76 publications, notably more studies than in earlier reviews and by covering 45 years from 1985 to 2020.

Twenty-eight specific correlates were investigated in this body of literature, although more than a quarter of them were only studied once. The widespread use of the case-control design makes all the study findings vulnerable to selection and information biases, as well as confounding [133–135] problems that can be mitigated by adequate sample sizes, strategies to minimize classification error, and recruitment of subjects representative of the pediatric self-harm population. Unfortunately, many of these studies fall short on one or more of these features. Conversely, studies which did have similar methods and were rated as Good did report similar findings, e.g., [89, 91, 94, 95].

Resolution of inter-study divergence in findings is challenging because of methodological heterogeneity on multiple levels: classification of self-harm; classification of subjects with respect to psychiatric patient status; an assortment of different types of controls and a surprising lack of uniformity in measuring the actual correlates. The use of multiple different instruments to classify subjects also undermines our ability to use these studies for biomarker development. Moreover, recent reviews of child and adolescent self-harm instruments have questioned their psychometric properties [136–138] and pointed out possible threats to validity when an instrument is used for purposes other than originally designed. Other data from adults demonstrate that 40% of those responding yes to a question about attempting suicide later denied the report [139]. This suggests that single questions may be misleading, but numerous studies did classify subjects with one or two questions.

Similar issues arose in the measurement of correlates or outcomes. Confounding was rarely handled by standard methods such as stratification or propensity scores [140] and researchers sometimes measured unique outcomes of specific correlates, making inter-study comparisons or interpretation of different findings challenging.

Although the patient samples from earlier studies may have been representative of self-harming children and adolescents in the 1980s and early 1990s, current information suggests that this is no longer the case. Up to 60% of adolescents with NSSI in the general population do not seek care [141] and half of the adolescents with suicidality or NSSI in a population study do not present for help [142]. Moreover, the ability to access care can be compromised by low socioeconomic status [143], rural geographic location [144], or minority race/ethnicity [145], thus reducing generalizability of findings for those who experience healthcare disparities. Similarly, recruiting participants based on a psychiatric disorder limits applicability of results to the sub-population of self-harming children or adolescents with that disorder, despite evidence that self-harm can be transdiagnostic or exist independent of psychiatric disorders [146–148].

We identified four research gaps: 1) the absence of replication studies; 2) a dearth of studies on children younger than 11 years old; 3) relatively few studies on non-patient children or adolescents, and 4) disproportionate representation of girls. A possible gap is the lack of data on non-white children and adolescents, but we could not confirm this.

If left unfilled, these gaps will significantly impede progress in this field. Replication studies can help verify that an association between self-harm and a specific correlate is not a spurious finding and they are a critical step in the development of all types of biomarkers [149]. Thus, they should be included in future research about pediatric peripheral and neural correlates of self-harm. These can be guided by some innovative research to determine which studies in a body of work should undergo replication [150–152].

More studies on correlates of self-harm in younger children are needed, as self-harm is increasing in younger age groups [153, 154]. For example, presentations to US emergency departments for suicidality increased substantially from 2007 to 2015 (the most recent data available) and 43% of those visits were from children 5–10 years of age. Moreover, suicide was the third leading cause of death in the US for younger children (10–12 years of age) (https://webappa.cdc.gov/sasweb/ncipc/leadcause.html) and the characteristics of younger children with self-harm are different from adolescents [5].

Gender proportions are essential to balance in the research landscape. Girls are more likely to engage in suicidality and NSSI, so samples comprised mostly or entirely of females can be appropriate. But results from such studies cannot be generalized to boys. Furthermore, given gender differences in help-seeking, it is unlikely that many boys with self-harm will be found in clinical settings.

New studies must increase the number of non-patient children and adolescents under investigation. It is also essential that samples are not only more diverse with regards to gender, but also for race and ethnicity, as recent data show that from 1991 to 2017 suicide attempts among black adolescents in the US rose 73%, compared to a decrease of 7.5% in white adolescents [155]. The current body of work is ill-suited to help us understand self-harm in black children and adolescents.

There were several strengths in this body of research, including a larger number of studies and a longer list of specific correlates than we expected to find based on previous reviews. In addition, the cohort and pre-post treatment studies provide good foundations for the development of prognostic and treatment biomarkers.

Our original goal was to prepare for a systematic review and meta-analysis in service of identifying correlates with potential for biomarker development. Clinical biomarker development requires that a representative and valid sample of the target population is studied with a feasible and standardized process for biomarker data collection and processing, and that replicability of results is shown in appropriate sub-populations [156, 157]. After these criteria have been satisfied, characteristics of the marker such as sensitivity, specificity, PPV, and NPV [158] must be established. Progression to biomarker development is not possible for the peripheral or neural correlates identified in our review, due to the small numbers of studies, concerns about self-harm classification, variability of findings, and methodologic weaknesses in measuring some of the specific correlates.

But this body of work could serve as an excellent platform for biomarker discovery if four improvements are made in future research. The first and most important pertains to the classification of self-harm. In the early to mid-2000s, there was widespread discussion of whether suicidality and NSSI lay on a continuum, i.e., with a predictable pattern of progression from NSSI to suicidal behavior or on a spectrum, i.e., co-occurring disorders that partially overlapped in characteristics and etiology, but comprising distinct clinical syndromes. The concept of a spectrum gained momentum, culminating in the US with the designation of NSSI and suicidal behavior disorder as separate disorders in need of further study in psychiatry’s Diagnostic and Statistical Manual (DSM)- 5 [159].

However, this approach may be difficult to use, based as it is in self-report about intention to die when engaging in self-harm. Some researchers assert this two-category conceptualization of self-harm has been inadequately validated [160], with concerns that investigations based on this schema will lead to invalid phenotyping [161, 162, 163, 164, 165].

To continue to acquire knowledge about correlates of self-harm in children and adolescents despite disagreements about the phenomenology, we recommend improving participant classification methods. Studies should collect and publish information about all types of self-harm, even if the study aim is to focus on one type. Optimizing the chance that homogeneous samples will be created if that is a goal, publishing results of this classification strategy will also deepen our understanding of the complex symptoms and behaviours comprising child and adolescent self-harm.

To increase the validity of classification, instruments with good psychometric properties in children and adolescents should be used. Approaches using one or two questions from instruments measuring other constructs are not recommended [166]. Furthermore, as the type of instrument, e.g., self-report checklist vs. clinician-rated instruments, can produce different prevalences of self-harm [167], we recommend classifying self-harm in subjects based on a transparent integration of data from several types of instruments [168]. We also recommend more research on the use of cognitive tasks (instead of, or in addition to self-report) to classify self-harm in children and adolescents, especially in younger children [169].

The second set of improvements involves minimizing bias in future correlate studies. All the methodologic issues in design, sample construction, correlate and outcome measurement discussed in this review are well-described in the epidemiologic literature. However, to ensure that bias and measurement errors are maximally mitigated, we suggest that researchers use one of the risk of bias instruments as a planning guide in the study development phase [170].

Advancement in this field will be stalled unless measurement of specific correlates and outcomes is standardized. Researchers working in each specific correlate area could substantially improve the capacity to detect associations between self-harm and correlates if there was agreement on the measurement of peripheral and neural correlates and their outcomes. Such a practice would also minimize chances that information bias or measurement error has produced low inter-study agreement in results.

Most of the 28 specific correlates investigated in our dataset were derived from research on adults. Our fourth recommendation is to encourage future researchers to use innovative strategies to search for new potential correlates in children and adolescents with self-harm. One possible source of new correlates is post-mortem studies of completed suicide in the pediatric age range. No post-mortem studies met inclusion criteria for this review. But studies modeled after the pioneering work of Pandey and colleagues [171] need to be conducted in 3–19-year-olds who have completed suicide. Another source of possible correlates are genome-wide association studies of persons who have completed suicide [172] and of those with other types of self-harm [173] or NSSI [174]. The burgeoning field of “omics” research beyond genomics is likely to be useful in generating possible correlates for investigation [175], whether studies are conducted on small samples of individuals [176] or use “ome-wide” data from population samples [177]. The work to date has primarily been done in adults, but we encourage researchers to apply the same strategies in 3–19-year-olds [178].

A final approach to identify new potential correlates or biomarkers is the use of machine learning, either with electronic health record (EHR) data [179] or in analyzing neural signatures in response to cognitive tasks [180]. There are many reservations about the use of these new approaches [181, 182], but as the machine learning field matures, strategies such as these may provide promising leads.

### Limitations

While having several strengths, our review also has limitations. First, we only obtained papers written in English, so may have missed important studies on the topic not written in English. Second, our search used only two databases, PubMed and Embase. However, these two cover medical and biomedical research from 1947 to the present, including Medline, conference abstracts, ebooks, and citations in non-medical journals. PubMed has 25 million records, while Embase has 29 million. Therefore, we do not think this search strategy missed studies, but it is possible. Third, we did not search the gray literature, nor did we write to prominent authors looking for unpublished studies, especially those with negative findings. Publication bias thus might explain why nearly every study in our dataset reported some association between self-harm and the specific correlate under investigation. Fourth, our categorization of self-harm studies was based on how investigators described their populations of interest or samples. Our classification system was too high-level for us to report on the more nuanced features of suicidality, e.g., suicidal plans, ideation, attempts or on specific NSSI behaviors, e.g. cutting or burning. Future researchers will likely want more detail on specific behavioral manifestations, but if so, such details are supplied in Supplements 3 and 4. Fifth, our assessments of risk of bias showed only moderate inter-rater agreement. The methodologic problems that we summarized in the studies are easy to list from questions asked in the rating process, but we are less confident about the qualitative ratings. Any future systematic reviews should ensure better agreement from the beginning of the process with better training or by using more quantitative rating systems.

## Conclusions

Our scoping review demonstrates that this corpus of research is not sufficiently mature for a meta-analysis to identify potential biomarkers. Many conflicting results are reported for the 28 specific correlates. Interpretation of the divergent results is hampered by methods that may have produced biased findings and samples mainly generalizable to clinical populations and girls. Most of the work was done in adolescents, not children younger than 11 years. Although the current research is not robust enough to identify potential biomarkers, it provides a platform for the next level of work. Our suggestions to improve future research should significantly advance the field and help promote biomarker development for the diagnosis, prognosis, and treatment of the growing problem of child and adolescent self-harm.

## Supplementary Information

**Additional file 1.** Preferred Reporting Items for Systematic reviews and Meta-Analyses extension for Scoping Reviews (PRISMA-ScR) Checklist.

**Additional file 2.** Search Strategy: PubMed & Embase, January 1, 1980-May 6, 2020.

**Additional file 3.** Study details for peripheral correlates for self-harm in children and adolescents.

**Additional file 4. **Study details for neural correlates of self-harm in children and adolescents.

## Data Availability

Data sharing is not applicable to this article as no datasets were generated or analysed during the study.

## References

[1] Lim KS, Wong CH, McIntyre RS, Wang J, Zhang Z, Tran BX, et al. Global lifetime and 12-month prevalence of suicidal behavior, deliberate self-harm and non-suicidal self-Injury in children and adolescents between 1989 and 2018: a meta-analysis. Int J Environ Res Public Health. 2019;16(22):4581. 10.3390/ijerph16224581.

[2] Suicide worldwide in 2019: Global Health Estimates [https://www.who.int/publications/i/item/9789240026643].

[3] State of Child Health [https://stateofchildhealth.rcpch.ac.uk/evidence/mortality/adolescent-mortality/].

[4] Bould H, Mars B, Moran P, Biddle L, Gunnell D (2019). Rising suicide rates among adolescents in England and Wales. Lancet.

[5] Sheftall AH, Asti L, Horowitz LM, Felts A, Fontanella CA, Campo JV, et al. Suicide in elementary school-aged children and early adolescents. Pediatrics. 2016;138(4):e20160436. 10.1542/peds.2016-0436.

[6] Zainum K, Cohen MC (2017). Suicide patterns in children and adolescents: a review from a pediatric institution in England. Forensic Sci Med Pathol.

[7] Borschmann R, Mundy LK, Canterford L, Moreno-Betancur M, Moran PA, Allen NB, et al. Self-harm in primary school-aged children: prospective cohort study. PLoS One. 2020;15(11):e0242802. 10.1371/journal.pone.0242802.

[8] Studart-Botto P, Martins-Junior DF, Sarmento S, Argolo L, Galvao-de-Almeida A, Miranda-Scippa A (2020). Self-injurious behavior and related mortality in children under 10 years of age: a retrospective health record study in Brazil. Braz J Psychiatry.

[9] Horowitz L, Tipton MV, Pao M (2020). Primary and secondary prevention of youth suicide. Pediatrics.

[10] Sveticic J, De Leo D. The hypothesis of a continuum in suicidality: a discussion on its validity and practical implications. Ment Illn. 2012;4(2):e15. 10.4081/mi.2012.e15.

[11] Olfson M, Wall M, Wang S, Crystal S, Bridge JA, Liu SM, et al. Suicide after deliberate self-harm in adolescents and young adults. Pediatrics. 2018;141(4):e20173517. 10.1542/peds.2017-3517.

[12] Stewart JG, Esposito EC, Glenn CR, Gilman SE, Pridgen B, Gold J, et al. Adolescent self-injurers: comparing non-ideators, suicide ideators, and suicide attempters. J Psychiatr Res. 2017;84:105-112. 10.1016/j.jpsychires.2016.09.031.

[13] Whitlock J, Knox KL (2007). The relationship between self-injurious behavior and suicide in a young adult population. Arch Pediatr Adolesc Med.

[14] Zetterqvist M, Lundh L, Dahlström O, Svedin C (2013). Prevalence and function of non-suicidal self-injury (NSSI) in a community sample of adolescents, using suggested DSM-5 criteria for a potential NSSI disorder. J Abnorm Child Psychol.

[15] Thippaiah M, Nanjappa S, Gude J, Voyiaziakis E, Patwa S, Birur B, Pandurangi A (2021). Non-suicidal self-injury in developing countries: a review. Int J Soc Psychiatry.

[16] Barrocas AL, Hankin BL, Young JF, Abela JR (2012). Rates of nonsuicidal self-injury in youth: age, sex, and behavioral methods in a community sample. Pediatrics.

[17] Luby JL, Whalen D, Tillman R, Barch DM (2019). Clinical and psychosocial characteristics of Young children with suicidal ideation, behaviors, and nonsuicidal self-injurious behaviors. J Am Acad Child Adolesc Psychiatry.

[18] Muehlenkamp JJ, Xhunga N, Brausch AM (2019). Self-injury age of onset: a risk factor for NSSI severity and suicidal behavior. Arch Suicide Res.

[19] Gardner W, Pajer K, Cloutier P, Currie L, Colman I, Zemek R, et al. Health outcomes associated with emergency department visits by adolescents for self-harm: a propensity-matched cohort study. CMAJ. 2019;191(44):e1207-e1216. 10.1503/cmaj.190188.

[20] Mars B, Heron J, Crane C, Hawton K, Lewis G, Macleod J, et al. Clinical and social outcomes of adolescent self harm: population based birth cohort study. BMJ. 2014;349:g5954. 10.1136/bmj.g5954.

[21] Courtney DB, Duda S, Szatmari P, Henderson J, Bennett K (2019). Systematic review and quality appraisal of practice guidelines for self-harm in children and adolescents. Suicide Life Threat Behav.

[22] Bahji A, Pierce M, Wong J, Roberge JN, Ortega I, Patten S. Comparative efficacy and acceptability of psychotherapies for self-harm and suicidal behavior among children and adolescents: a systematic review and network meta-analysis. JAMA Netw Open. 2021;4(4):e216614. 10.1001/jamanetworkopen.

[23] Witt KG, Hetrick SE, Rajaram G, Hazell P, Taylor Salisbury TL, Townsend E, et al. Interventions for self-harm in children and adolescents. Cochrane Database Syst Rev. 2021;3:Cd013667. 10.1002/14651858.CD012013.

[24] Kothgassner OD, Robinson K, Goreis A, Ougrin D, Plener PL. Does treatment method matter? A meta-analysis of the past 20 years of research on therapeutic interventions for self-harm and suicidal ideation in adolescents. Borderline Personal Disord Emot Dysregulation. 2020;7:9. 10.1186/s40479-020-00123-9.

[25] Russell DH, Trew S, Higgins DJ (2021). Vulnerable yet forgotten? A systematic review identifying the lack of evidence for effective suicide interventions for young people in contact with child protection systems. Am J Orthop.

[26] Hawton K, Witt KG, Taylor Salisbury TL, Arensman E, Gunnell D, Hazell P, et al. Pharmacological interventions for self-harm in adults. Cochrane Database Syst Rev. 2015;7:CD011777. 10.1002/14651858.CD011777.

[27] Hawton K, Saunders KE, O'Connor RC (2012). Self-harm and suicide in adolescents. Lancet.

[28] King J, Cabarkapa S, Leow F (2019). Adolescent self-harm: think before prescribing. Aust Prescr.

[29] Garcia-Gutierrez MS, Navarrete F, Sala F, Gasparyan A, Austrich-Olivares A, Manzanares J. Biomarkers in psychiatry: concept, definition, types and relevance to the clinical reality. Front Psychiatry. 2020;11:432. 10.3389/fpsyt.2020.00432.

[30] Mathew A, Nebhinani N, Begum S, Vijayakumar K (2021). M B: genetics of adolescent suicide: a literature review. J Indian Assoc Child Adolesc Ment Health.

[31] Xiang C, Liu S, Fan Y, Wang X, Jia Y, Li L, et al. Single nucleotide polymorphisms, variable number tandem repeats and allele influence on serotonergic enzyme modulators for aggressive and suicidal behaviors: a review. Pharmacol Biochem Behav. 2019;180:74–82. 10.1016/j.pbb.2019.03.008.

[32] Mirkovic B, Laurent C, Podlipski MA, Frebourg T, Cohen D, Gerardin P. Genetic association studies of suicidal behavior: a review of the past 10 years, progress, limitations, and future directions. Front Psychiatry. 2016;7:158. 10.3389/fpsyt.2016.00158.

[33] van Heeringen K, Bijttebier S, Desmyter S, Vervaet M, Baeken C. Is there a neuroanatomical basis of the vulnerability to suicidal behavior? A coordinate-based meta-analysis of structural and functional MRI studies. Front Hum Neurosci. 2014;8:824. 10.3389/fnhum.2014.00824.

[34] Kim JW, Szigethy EM, Melhem NM, Saghafi EM, Brent DA (2014). Inflammatory markers and the pathogenesis of pediatric depression and suicide: a systematic review of the literature. J Clin Psychiatry.

[35] Cha CB, Franz PJ, Guzman EM, Glenn CR, Kleiman EM, Nock MK. Annual research review: suicide among youth - epidemiology, (potential) etiology, and treatment. J Child Psychol Psychiatry. 2018;59(4):460–82.

[36] Lewitzka U, Doucette S, Seemuller F, Grof P, Duffy AC (2012). Biological indicators of suicide risk in youth with mood disorders: what do we know so far?. Curr Psychiatry Rep.

[37] Picouto MD, Villar F, Braquehais MD (2015). The role of serotonin in adolescent suicide: theoretical, methodological, and clinical concerns. Int J Adolesc Med Health.

[38] Martin PC, Zimmer TJ, Pan LA (2015). Magnetic resonance imaging markers of suicide attempt and suicide risk in adolescents. CNS Spectr.

[39] Groschwitz R, Plener P (2012). The neurobiology of non-suicidal self-injury (NSSI): a review. Suicidology Online.

[40] Auerbach RP, Pagliaccio D, Allison GO, Alqueza KL, Alonso MF (2021). Neural correlates associated with suicide and nonsuicidal self-injury in youth. Biol Psychiatry.

[41] Kaess M, Hooley JM, Klimes-Dougan B, Koenig J, Plener PL, Reichl C, et al. Advancing a temporal framework for understanding the biology of nonsuicidal self- injury: an expert review. Neurosci Biobehav Rev. 2021;130:228–39. 10.1016/j.neubiorev.2021.08.022.

[42] Safer DJ (1997). Adolescent/adult differences in suicidal behavior and outcome. Ann Clin Psychiatry.

[43] Hagan CR, Rogers ML, Brausch AM, Muehlenkamp JJ, Joiner TE (2019). Interoceptive deficits, non-suicidal self-injury, and suicide risk: a multi-sample study of indirect effects. Psychol Med.

[44] Lee J, Bang YS, Min S, Ahn JS, Kim H, Cha YS, et al. Characteristics of adolescents who visit the emergency department following suicide attempts: comparison study between adolescents and adults. BMC Psychiatry. 2019;19(1):231. 10.1186/s12888-019-2213-5.

[45] Shain B, Committee on Adolescence. Suicide and suicide attempts in adolescents. Pediatrics. 201;138(1):e20161420. 10.1542/peds.2016-1420.

[46] Munn Z, Peters MDJ, Stern C, Tufanaru C, McArthur A, Aromataris E. Systematic review or scoping review? Guidance for authors when choosing between a systematic or scoping review approach. BMC Med Res Methodol. 2018;18(1):143. 10.1186/s12874-018-0611-x.

[47] Peters MDJ, Godfrey C, McInerney P, Munn Z, Tricco AC, Khalil, H. Chapter 11: Scoping Reviews (2020 version). In: Aromataris E, Munn Z (Editors). JBI Manual for Evidence Synthesis, JBI, 2020. https://synthesismanual.jbi.global, 10.46658/JBIMES-20-12.

[48] Tricco AC, Lillie E, Zarin W, O'Brien K, Colquhoun H, Levac D, Moher D, Peters MDJ, Ma Q, Horsley T (2018). PRISMA extension for scoping reviews ( PRISMA-ScR ): checklist and explanation. Ann Intern Med.

[49] Covidence systematic review software [https://www.covidence.org/].

[50] The Strengthening the Reporting of Observational Studies in Epidemiology (STROBE) Statement: guidelines for reporting observational studies [https://www.equator-network.org/reporting-guidelines/strobe/].

[51] Crowell SE, Beauchaine TP, McCauley E, Smith CJ, Stevens AL, Sylvers P (2005). Psychological, autonomic, and serotonergic correlates of parasuicide among adolescent girls. Dev Psychopathol.

[52] Falcone T, Fazio V, Lee C, Simon B, Franco K, Marchi N, et al. Serum S100B: a potential biomarker for suicidality in adolescents? PLoS One. 2010;5(6):e11089. 10.1371/journal.pone.0011089.

[53] Pan LA, Hassel S, Segreti AM, Nau SA, Brent DA, Phillips ML (2013). Differential patterns of activity and functional connectivity in emotion processing neural circuitry to angry and happy faces in adolescents with and without suicide attempt. Psychol Med.

[54] Robbins DR, Alessi NE (1985). Suicide and the dexamethasone suppression test in adolescence. Biol Psychiatry.

[55] Rosenthal PA, Rosenthal S, Doherty MB, Santora D (1986). Suicidal thoughts and behaviors in depressed hospitalized preschoolers. Am J Psychother.

[56] Dahl RE, Ryan ND, Puig-Antich J (1991). Nguyen NA, al-Shabbout M, Meyer VA, Perel J: 24-hour cortisol measures in adolescents with major depression: a controlled study. Biol Psychiatry.

[57] Dahl RE, Kaufman J, Ryan ND (1992). Perel J, al-Shabbout M, Birmaher B, Nelson B, Puig-Antich J: the dexamethasone suppression test in children and adolescents: a review and a controlled study. Biol Psychiatry.

[58] Ghaziuddin N, King CA, Welch K, Ghaziuddin M (2014). Depressed suicidal adolescent males have an altered cortisol response to a pharmacological challenge. Asian J Psychiatr.

[59] Young R (2010). Trauma, attempted suicide, and morning cortisol in a community sample of adolescents. J Trauma Stress.

[60] Pfeffer CR, Stokes P, Shindledecker R (1991). Suicidal behavior and hypothalamic-pituitary-adrenocortical axis indices in child psychiatric inpatients. Biol Psychiatry.

[61] Giletta M, Calhoun CD, Hastings PD, Rudolph KD, Nock MK, Prinstein MJ (2015). Multi-level risk factors for suicidal ideation among at-risk adolescent females: the role of hypothalamic-pituitary-adrenal axis responses to stress. J Abnorm Child Psychol.

[62] Eisenlohr-Moul TA, Miller AB, Giletta M, Hastings PD, Rudolph KD, Nock MK, Prinstein MJ (2018). HPA axis response and psychosocial stress as interactive predictors of suicidal ideation and behavior in adolescent females: a multilevel diathesis-stress framework. Neuropsychopharmacology.

[63] Reichl C, Heyer A, Brunner R, Parzer P, Völker JM, Resch F, Kaess M (2016). Hypothalamic-pituitary-adrenal axis, childhood adversity and adolescent nonsuicidal self-injury. Psychoneuroendocrinology.

[64] Klimes-Dougan B, Begnel E, Almy B, Thai M, Schreiner MW, Cullen KR. Hypothalamic-pituitary-adrenal axis dysregulation in depressed adolescents with non-suicidal self-injury. Psychoneuroendocrinology. 2019;102:216-224. 10.1016/j.psyneuen.2018.11.004.

[65] Reichl C, Brunner R, Bender N, Parzer P, Koenig J, Resch F, et al. Adolescent nonsuicidal self-injury and cortisol response to the retrieval of adversity: a sibling study. Psychoneuroendocrinology. 2019;110:104460. 10.1016/j.psyneuen.2019.104460.

[66] Beauchaine TP, Crowell SE, Hsiao RC (2015). Post-dexamethasone cortisol, self-inflicted injury, and suicidal ideation among depressed adolescent girls. J Abnorm Child Psychol.

[67] Plener PL, Zohsel K, Hohm E, Buchmann AF, Banaschewski T, Zimmermann US, Laucht M (2016). Lower cortisol level in response to a psychosocial stressor in young females with self-harm. Psychoneuroendocrinology.

[68] Yang X, Daches S, George CJ, Kiss E, Kapornai K, Baji I, et al. Autonomic correlates of lifetime suicidal thoughts and behaviors among adolescents with a history of depression. Psychophysiology. 2019;56(8):e13378. 10.1111/psyp.13378.

[69] Giletta M, Hastings PD, Rudolph KD, Bauer DJ, Nock MK, Prinstein MJ (2017). Suicide ideation among high-risk adolescent females: examining the interplay between parasympathetic regulation and friendship support. Dev Psychopathol.

[70] Koenig J, Rinnewitz L, Parzer P, Resch F, Thayer JF, Kaess M. Resting cardiac function in adolescent non-suicidal self-injury: the impact of borderline personality disorder symptoms and psychosocial functioning. Psychiatry Res. 2017;248:117-120. 10.1016/j.psychres.2016.12.024.

[71] Crowell SE, Beauchaine TP, Hsiao RC, Vasilev CA, Yaptangco M, Linehan MM, McCauley E (2012). Differentiating adolescent self-injury from adolescent depression: possible implications for borderline personality development. J Abnorm Child Psychol.

[72] Wielgus MD, Aldrich JT, Mezulis AH, Crowell SE. Respiratory sinus arrhythmia as a predictor of self-injurious thoughts and behaviors among adolescents. Int J Psychophysiol. 2016;106:127-134. 10.1016/j.ijpsycho.2016.05.005.

[73] Aldrich JT, Wielgus MD, Mezulis AH. Low physiological arousal and high impulsivity as predictors of self-injurious thoughts and behaviors among adolescents. J Adolesc. 2018;62:55-60. 10.1016/j.adolescence.2017.11.006.

[74] Kaess M, Hille M, Parzer P, Maser-Gluth C, Resch F, Brunner R (2012). Alterations in the neuroendocrinological stress response to acute psychosocial stress in adolescents engaging in nonsuicidal self-injury. Psychoneuroendocrinology.

[75] Koenig J, Rinnewitz L, Warth M, Hillecke TK, Brunner R, Resch F, Kaess M (2017). Psychobiological response to pain in female adolescents with nonsuicidal self-injury. J Psychiatry Neurosci.

[76] Modai I, Apter A, Meltzer M, Tyano S, Walevski A, Jerushalmy Z (1989). Serotonin uptake by platelets of suicidal and aggressive adolescent psychiatric inpatients. Neuropsychobiology.

[77] Ambrosini PJ, Metz C, Arora RC, Lee JC, Kregel L, Meltzer HY (1992). Platelet imipramine binding in depressed children and adolescents. J Am Acad Child Adolesc Psychiatry.

[78] Pfeffer CR, McBride PA, Anderson GM, Kakuma T, Fensterheim L, Khait V (1998). Peripheral serotonin measures in prepubertal psychiatric inpatients and normal children: associations with suicidal behavior and its risk factors. Biol Psychiatry.

[79] Tyano S, Zalsman G, Ofek H, Blum I, Apter A, Wolovik L, Sher L, Sommerfeld E, Harell D, Weizman A (2006). Plasma serotonin levels and suicidal behavior in adolescents. Eur Neuropsychopharmacol.

[80] Pine DS, Trautman PD, Shaffer D, Cohen L, Davies M, Stanley M, Parsons B (1995). Seasonal rhythm of platelet [3H] imipramine binding in adolescents who attempted suicide. Am J Psychiatry.

[81] Clark DB (2003). Serum tryptophan ratio and suicidal behavior in adolescents: a prospective study. Psychiatry Res.

[82] Crowell SE, Beauchaine TP, McCauley E, Smith CJ, Vasilev CA, Stevens AL (2008). Parent-child interactions, peripheral serotonin, and self-inflicted injury in adolescents. J Consult Clin Psychol.

[83] Dahl RE, Puig-Antich J, Ryan ND, Nelson B, Dachille S, Cunningham SL, Trubnick L, Klepper TP (1990). EEG sleep in adolescents with major depression: the role of suicidality and inpatient status. J Affect Disord.

[84] Dahl RE, Ryan ND (1991). Birmaher B, al-Shabbout M, Williamson DE, Neidig M, Nelson B, Puig-Antich J: electroencephalographic sleep measures in prepubertal depression. Psychiatry Res.

[85] Emslie GJ, Rush AJ, Weinberg WA, Rintelmann JW, Roffwarg HP (1994). Sleep EEG features of adolescents with major depression. Biol Psychiatry.

[86] McCracken JT, Poland RE, Lutchmansingh P, Edwards C (1997). Sleep electroencephalographic abnormalities in adolescent depressives: effects of scopolamine. Biol Psychiatry.

[87] Boafo A, Armitage R, Greenham S, Tavakoli P, Dale A, Nixon A, et al. Sleep architecture in adolescents hospitalized during a suicidal crisis. Sleep Med. 2019;56:41-46. 10.1016/j.sleep.2018.12.018.

[88] Singareddy R, Krishnamurthy VB, Vgontzas AN, Fernandez-Mendoza J, Calhoun SL, Shaffer ML, Bixler EO (2013). Subjective and objective sleep and self-harm behaviors in young children: a general population study. Psychiatry Res.

[89] Bilgiç A, Çelikkol Sadıç Ç, Kılınç İ (2020). Akça Ö F: exploring the association between depression, suicidality and serum neurotrophin levels in adolescents. Int J Psychiatry Clin Pract.

[90] Falcone T, Janigro D, Lovell R, Simon B, Brown CA, Herrera M, Myint AM, Anand A (2015). S100B blood levels and childhood trauma in adolescent inpatients. J Psychiatr Res.

[91] Kavurma C, Tas FV, Demirgoren BS, Demirci F, Akan P, Eyuboglu D, et al. Do serum BDNF levels vary in self-harm behavior among adolescents and are they correlated with traumatic experiences? Psychiatry Res. 2017;258:130-5. 10.1016/j.psychres.2017.09.069.

[92] Gabbay V, Klein RG, Guttman LE, Babb JS, Alonso CM, Nishawala M, Katz Y, Gaite MR, Gonzalez CJ (2009). A preliminary study of cytokines in suicidal and nonsuicidal adolescents with major depression. J Child Adolesc Psychopharmacol.

[93] Amitai M, Taler M, Ben-Baruch R, Lebow M, Rotkopf R, Apter A, et al. Increased circulatory IL-6 during 8-week fluoxetine treatment is a risk factor for suicidal behaviors in youth. Brain Behav Immun. 2020;87:301-8. 10.1016/j.bbi.2019.12.017.

[94] Glueck CJ, Kuller FE, Hamer T, Rodriguez R, Sosa F, Sieve-Smith L, Morrison JA (1994). Hypocholesterolemia, hypertriglyceridemia, suicide, and suicide ideation in children hospitalized for psychiatric diseases. Pediatr Res.

[95] Plana T, Gracia R, Méndez I, Pintor L, Lazaro L, Castro-Fornieles J (2010). Total serum cholesterol levels and suicide attempts in child and adolescent psychiatric inpatients. Eur Child Adolesc Psychiatry.

[96] Ryan ND, Puig-Antich J, Rabinovich H, Ambrosini P, Robinson D, Nelson B, Novacenko H (1988). Growth hormone response to desmethylimipramine in depressed and suicidal adolescents. J Affect Disord.

[97] Dahl RE, Ryan ND, Williamson DE, Ambrosini PJ, Rabinovich H, Novacenko H, Nelson B, Puig-Antich J (1992). Regulation of sleep and growth hormone in adolescent depression. J Am Acad Child Adolesc Psychiatry.

[98] Pan LA, Batezati-Alves SC, Almeida JR, Segreti A, Akkal D, Hassel S, et al. Dissociable patterns of neural activity during response inhibition in depressed adolescents with and without suicidal behavior. J Am Acad Child Adolesc Psychiatry. 2011;50(6):602–611.e3. 10.1016/j.jaac.2011.03.018.

[99] Pan L, Segreti A, Almeida J, Jollant F, Lawrence N, Brent D, Phillips M (2013). Preserved hippocampal function during learning in the context of risk in adolescent suicide attempt. Psychiatry Res.

[100] Quevedo K, Ng R, Scott H, Martin J, Smyda G, Keener M, Oppenheimer CW (2016). The neurobiology of self-face recognition in depressed adolescents with low or high suicidality. J Abnorm Psychol.

[101] Harms MB, Casement MD, Teoh JY, Ruiz S, Scott H, Wedan R, et al. Adolescent suicide attempts and ideation are linked to brain function during peer interactions. Psychiatry Res Neuroimaging. 2019;289:1–9. 10.1016/j.pscychresns.2019.05.001.

[102] Oppenheimer CW, Silk JS, Lee KH, Dahl RE, Forbes E, Ryan N, Ladouceur CD (2020). Suicidal ideation among anxious youth: a preliminary investigation of the role of neural processing of social rejection in interaction with real world negative social experiences. Child Psychiatry Hum Dev.

[103] Plener PL, Bubalo N, Fladung AK, Ludolph AG, Lulé D (2012). Prone to excitement: adolescent females with non-suicidal self-injury (NSSI) show altered cortical pattern to emotional and NSS-related material. Psychiatry Res.

[104] Groschwitz RC, Plener PL, Groen G, Bonenberger M, Abler B. Differential neural processing of social exclusion in adolescents with non-suicidal self-injury: an fMRI study. Psychiatry Res Neuroimaging. 2016;255:43–9. 10.1016/j.pscychresns.2016.08.001.

[105] Quevedo K, Martin J, Scott H, Smyda G, Pfeifer JH. The neurobiology of self-knowledge in depressed and self-injurious youth. Psychiatry Res Neuroimaging. 2016;254:145–55. 10.1016/j.pscychresns.2016.06.015.

[106] Brown RC, Plener PL, Groen G, Neff D, Bonenberger M, Abler B. Differential neural processing of social exclusion and inclusion in adolescents with non-suicidal self-injury and Young adults with borderline personality disorder. Front Psychiatry. 2017;8:267. 10.3389/fpsyt.2017.00267.

[107] Perini I, Gustafsson PA, Hamilton JP, Kampe R, Mayo LM, Heilig M, et al. Brain-based classification of negative social bias in adolescents with nonsuicidal self-injury: findings from simulated online social interaction. EClinicalMedicine. 2019;13:81-90. 10.1016/j.eclinm.2019.06.016.

[108] Poon JA, Thompson JC, Forbes EE, Chaplin TM (2019). Adolescents' reward-related neural activation: links to thoughts of nonsuicidal self-injury. Suicide Life Threat Behav.

[109] Sauder CL, Derbidge CM, Beauchaine TP (2016). Neural responses to monetary incentives among self-injuring adolescent girls. Dev Psychopathol.

[110] Alarcón G, Sauder M, Teoh JY, Forbes EE, Quevedo K (2019). Amygdala functional connectivity during self-face processing in depressed adolescents with recent suicide attempt. J Am Acad Child Adolesc Psychiatry.

[111] Ordaz SJ, Goyer MS, Ho TC, Singh MK, Gotlib IH. Network basis of suicidal ideation in depressed adolescents. J Affect Disord. 2018;226:92-99. 10.1016/j.jad.2017.09.021.

[112] Schreiner MW, Klimes-Dougan B, Cullen KR (2019). Neural correlates of suicidality in adolescents with major depression: resting-state functional connectivity of the precuneus and posterior cingulate cortex. Suicide Life Threat Behav.

[113] Schwartz J, Ordaz SJ, Ho TC, Gotlib IH. Longitudinal decreases in suicidal ideation are associated with increases in salience network coherence in depressed adolescents. J Affect Disord. 2019;245:545-552. 10.1016/j.jad.2018.11.009.

[114] Santamarina-Perez P, Romero S, Mendez I, Leslie SM, Packer MM, Sugranyes G, Picado M, Font E, Moreno E, Martinez E (2019). Fronto-limbic connectivity as a predictor of improvement in nonsuicidal self-injury in adolescents following psychotherapy. J Child Adolesc Psychopharmacol.

[115] Tavakoli P, Boafo A, Dale A, Robillard R, Greenham SL, Campbell K. Event-related potential measures of attention capture in adolescent inpatients with acute suicidal behavior. Front Psychiatry. 2018;9:85. 10.3389/fpsyt.2018.00085.

[116] Tsypes A, Owens M, Gibb BE (2019). Blunted neural reward responsiveness in children with recent suicidal ideation. Clin Psychol Sci.

[117] Pegg S, Dickey L, Green H, Kujawa A (2020). Differentiating clinically depressed adolescents with and without active suicidality: an examination of neurophysiological and self-report measures of reward responsiveness. Depress Anxiety.

[118] Tsypes A, Owens M, Hajcak G, Gibb BE (2018). Neural reward responsiveness in children who engage in nonsuicidal self-injury: an ERP study. J Child Psychol Psychiatry.

[119] Graae F, Tenke C, Bruder G, Rotheram MJ, Piacentini J, Castro-Blanco D, Leite P, Towey J (1996). Abnormality of EEG alpha asymmetry in female adolescent suicide attempters. Biol Psychiatry.

[120] Lewis CP, Camsari DD, Sonmez AI, Nandakumar AL, Gresbrink MA, Daskalakis ZJ, et al. Preliminary evidence of an association between increased cortical inhibition and reduced suicidal ideation in adolescents treated for major depression. J Affect Disord. 2019;244:21–4. 10.1016/j.jad.2018.09.079.

[121] Ho TC, Cichocki AC, Gifuni AJ, Catalina Camacho M, Ordaz SJ, Singh MK, Gotlib IH (2018). Reduced dorsal striatal gray matter volume predicts implicit suicidal ideation in adolescents. Soc Cogn Affect Neurosci.

[122] Ando A, Reichl C, Scheu F, Bykova A, Parzer P, Resch F, et al. Regional grey matter volume reduction in adolescents engaging in non-suicidal self-injury. Psychiatry Res Neuroimaging. 2018;280:48-55. 10.1016/j.pscychresns.2018.08.005.

[123] Beauchaine TP, Sauder CL, Derbidge CM, Uyeji LL (2019). Self-injuring adolescent girls exhibit insular cortex volumetric abnormalities that are similar to those seen in adults with borderline personality disorder. Dev Psychopathol.

[124] Pan LA, Ramos L, Segreti A, Brent DA, Phillips ML (2015). Right superior temporal gyrus volume in adolescents with a history of suicide attempt. Br J Psychiatry.

[125] Goodman M, Hazlett EA, Avedon JB, Siever DR, Chu KW, New AS (2011). Anterior cingulate volume reduction in adolescents with borderline personality disorder and co-morbid major depression. J Psychiatr Res.

[126] Jovev M, Garner B, Phillips L, Velakoulis D, Wood SJ, Jackson HJ, Pantelis C, McGorry PD, Chanen AM (2008). An MRI study of pituitary volume and parasuicidal behavior in teenagers with first-presentation borderline personality disorder. Psychiatry Res.

[127] Achenbach T, Rescorla L (2001). Manual for the ASEBA School-age Forms & Profiles.

[128] Kaufman J, Birmaher B, Brent D, Rao U, Flynn C, Moreci P, Williamson D, Ryan N (1997). Schedule for affective disorders and schizophrenia for school-age children-present and lifetime version (K-SADS-PL): initial reliability and validity data. J Am Acad Child Adolesc Psychiatry.

[129] Nock MK, Holmberg EB, Photos VI, Michel BD (2007). Self-injurious thoughts and behaviors interview: development, reliability, and validity in an adolescent sample. Psychol Assess.

[130] Posner K, Brown GK, Stanley B, Brent DA, Yershova KV, Oquendo MA, et al. The Columbia-Suicide Severity Rating Scale: initial validity and internal consistency findings from three multisite studies with adolescents and adults. Am J Psychiatry. 2011;168(12):1266–77.

[131] Reynolds W. SIQ, suicidal ideation questionnaire: professional manual. Odessa, Florida: Psychological Assessment Resources; 1988.

[132] Altman D (1991). Practical statistics for medical research.

[133] Martinez D, Papuzinski C, Stojanova J, Arancibia M. General concepts in biostatistics and clinical epidemiology: observational studies with case-control design. Medwave. 2019;19(10):e7716. 10.5867/medwave.2019.10.7716.

[134] Wang X, Cheng Z (2020). Cross-sectional studies: strengths, weaknesses, and recommendations. Chest.

[135] Sedgwick P. Bias in observational study designs: case-control studies. BMJ. 2015;350:h560. 10.1136/bmj.h560.

[136] Carter T, Walker GM, Aubeeluck A, Manning JC (2019). Assessment tools of immediate risk of self-harm and suicide in children and young people: a scoping review. J Child Health Care.

[137] Harris IM, Beese S, Moore D. Predicting future self-harm or suicide in adolescents: a systematic review of risk assessment scales/tools. BMJ Open. 2019;9(9):e029311. 10.1136/bmjopen-2019-029311.

[138] Faura-Garcia J, Orue I, Calvete E (2021). Clinical assessment of non-suicidal self-injury: a systematic review of instruments. Clin Psychol Psychother.

[139] Nock MK, Kessler RC (2006). Prevalence of and risk factors for suicide attempts versus suicide gestures: analysis of the National Comorbidity Survey. J Abnorm Psychol.

[140] Kahlert J, Gribsholt SB, Gammelager H, Dekkers OM, Luta G. Control of confounding in the analysis phase - an overview for clinicians. Clin Epidemiol. 2017;9:195–204. 10.2147/CLEP.S129886.

[141] McManus S, Gunnell D, Cooper C, Bebbington PE, Howard LM, Brugha T, Jenkins R, Hassiotis A, Weich S, Appleby L (2019). Prevalence of non-suicidal self-harm and service contact in England, 2000-14: repeated cross-sectional surveys of the general population. Lancet Psychiatry.

[142] Steinhoff A, Ribeaud D, Kupferschmid S, Raible-Destan N, Quednow BB, Hepp U, et al. Self-injury from early adolescence to early adulthood: age-related course, recurrence, and services use in males and females from the community. Eur Child Adolesc Psychiatry. 2021;30(6):937-951.

[143] Hodgkinson S, Godoy L, Beers LS, Lewin A: Improving mental health access for low-income children and families in the primary care setting. Pediatrics. 2017;139(1):e20151175. 10.1542/peds.2015-1175.

[144] Morales DA, Barksdale CL, Beckel-Mitchener AC (2020). A call to action to address rural mental health disparities. J Clin Transl Sci.

[145] Whitney DG, Peterson MD (2019). US national and state-level prevalence of mental health disorders and disparities of mental health care use in children. JAMA Pediatr.

[146] Glenn CR, Klonsky ED (2013). Nonsuicidal self-injury disorder: an empirical investigation in adolescent psychiatric patients. J Clin Child Adolesc Psychol.

[147] Wetzler S, Asnis GM, Hyman RB, Virtue C, Zimmerman J, Rathus JH (1996). Characteristics of suicidality among adolescents. Suicide Life Threat Behav.

[148] Swannell S, Martin G, Scott J, Gibbons M, Gifford S (2008). Motivations for self-injury in an adolescent inpatient population: development of a self-report measure. Australas Psychiatry.

[149] McShane L (2017). In pursuit of greater reproducibility and credibility of early clinical biomarker research. Clin Transl Sci.

[150] Zwaan RA, Etz A, Lucas RE, Donnellan MB. Making replication mainstream. Behav Brain Sci. 2017;41:e120. 10.1017/S0140525X17001972.

[151] Coles NA, Tiokhin L, Scheel AM, Isager PM, Lakens D. The costs and benefits of replication studies. Behav Brain Sci. 2018;41:e124. 10.1017/S0140525X18000596.

[152] Field SM, Hoekstra R, Bringmann L, van Ravenzwaaij D. (When and why to replicate: As easy as 1, 2, 3? Collabra Psychol. 2019;5(1):Article 46. 10.1525/collabra.218.

[153] Ruch DA, Sheftall AH, Schlagbaum P, Rausch J, Campo JV, Bridge JA. Trends in suicide among youth aged 10 to 19 years in the United States, 1975 to 2016. JAMA Netw Open. 2019;2(5):e193886. 10.1001/jamanetworkopen.2019.3886.

[154] Griffin E, McMahon E, McNicholas F, Corcoran P, Perry IJ, Arensman E (2018). Increasing rates of self-harm among children, adolescents and young adults: a 10-year national registry study 2007-2016. Soc Psychiatry Psychiatr Epidemiol.

[155] Lindsey M, Sheftall A, Xiao Y, Joe S. Trends of suicidal behaviors among high school students in the United States: 1991-2017. Pediatrics. 2019;144(5):e20191187. 10.1542/peds.2019-1187.

[156] Scarr E, Millan MJ, Bahn S, Bertolino A, Turck CW, Kapur S, et al. Biomarkers for psychiatry: the journey from fantasy to fact, a report of the 2013 CINP think tank. Int J Neuropsychopharmacol. 2015, 18(10):pyv042. 10.1093/ijnp/pyv042.

[157] Abi-Dargham A, Horga G (2016). The search for imaging biomarkers in psychiatric disorders. Nat Med.

[158] Ray P, Le Manach Y, Riou B, Houle TT (2010). Statistical evaluation of a biomarker. Anesthesiology.

[159] American Psychiatric Association (2013). Diagnostic and statistical manual of mental disorders: DSM-5.

[160] Ghinea D, Edinger A, Parzer P, Koenig J, Resch F, Kaess M. Non-suicidal self-injury disorder as a stand-alone diagnosis in a consecutive help-seeking sample of adolescents. J Affect Disord. 2020;274:1122-5. 10.1016/j.jad.2020.06.009.

[161] Kapur N, Cooper J, O'Connor RC, Hawton K (2013). Non-suicidal self-injury v. attempted suicide: new diagnosis or false dichotomy?. Br J Psychiatry.

[162] Voss C, Hoyer J, Venz J, Pieper L, Beesdo-Baum K (2020). Non-suicidal self-injury and its co-occurrence with suicidal behavior: an epidemiological-study among adolescents and young adults. Acta Psychiatr Scand.

[163] Klonsky ED, Victor SE, Saffer BY (2014). Nonsuicidal self-injury: what we know, and what we need to know. Can J Psychiatr.

[164] Wilson E, Ougrin D. Commentary: Defining self-harm: how inconsistencies in language persist - a commentary/reflection on Ward and Curran (2021). Child Adolesc Ment Health. 2021;26(4):372-4.

[165] Huang X, Ribeiro J, Franklin J. The differences between individuals engaging in nonsuicidal self-injury and suicide attempt are complex (vs. complicated or simple). Front Psychiatry. 2020;11:239. 10.3389/fpsyt.2020.00239.

[166] Hom MA, Joiner TE, Bernert RA (2016). Limitations of a single-item assessment of suicide attempt history: implications for standardized suicide risk assessment. Psychol Assess.

[167] Muehlenkamp JJ, Claes L, Havertape L, Plener PL. International prevalence of adolescent non-suicidal self-injury and deliberate self-harm. Child Adolesc Psychiatry Ment Health. 2012;6:10. 10.1186/1753-2000-6-10.

[168] Deming CA, Harris JA, Castro-Ramirez F, Glenn JJ, Cha CB, Millner AJ, Nock MK (2021). Inconsistencies in self-reports of suicidal ideation and attempts across assessment methods. Psychol Assess.

[169] Mou D, Kleiman EM, Nock MK (2020). Proposed directions for suicide research: incorporating successful approaches from other disciplines. Br J Psychiatry.

[170] Page MJ, McKenzie JE, Higgins JPT. Tools for assessing risk of reporting biases in studies and syntheses of studies: a systematic review. BMJ Open. 2018;8(3):e019703. 10.1136/bmjopen-2017-019703.

[171] Pandey GN, Dwivedi Y. Neurobiology of teenage suicide. In: Dwivedi Y, editor. The neurobiological basis of suicide. Boca Raton, FL: CRC Press, 2012. pp.315-332.

[172] Docherty AR, Shabalin AA, DiBlasi E, Monson E, Mullins N, Adkins DE, Bacanu SA, Bakian AV, Crowell S, Chen D (2020). Genome-wide association study of suicide death and polygenic prediction of clinical antecedents. Am J Psychiatry.

[173] Strawbridge RJ, Ward J, Ferguson A, Graham N, Shaw RJ, Cullen B, Pearsall R, Lyall LM, Johnston KJA, Niedzwiedz CL (2019). Identification of novel genome-wide associations for suicidality in UK biobank, genetic correlation with psychiatric disorders and polygenic association with completed suicide. EBioMedicine.

[174] Campos AI, Verweij KJH, Statham DJ, Madden PAF, Maciejewski DF, Davis KAS, et al. Genetic aetiology of self-harm ideation and behaviour. Sci Rep. 2020;10(1):9713. 10.1038/s41598-020-66737-9.

[175] Kouter K, Paska AV: 'Omics' of suicidal behaviour: a path to personalised psychiatry. World J Psychiatry 2021;11(10):774–790.

[176] Mitro SD, Larrabure-Torrealva GT, Sanchez SE, Molsberry SA, Williams MA, Clish C, et al. Metabolomic markers of antepartum depression and suicidal ideation. J Affect Disord. 2020;262:422–8. 10.1016/j.jad.2019.11.061.

[177] Zacharias HU, Hertel J, Johar H, Pietzner M, Lukaschek K, Atasoy S, et al. A metabolome-wide association study in the general population reveals decreased levels of serum laurylcarnitine in people with depression. [published online ahead of print] Mol Psychiatry. 2021;10.1038/s41380-021-01176-0. 10.1038/s41380-021-01176-0.

[178] Simkin DR. Microbiome and mental health, specifically as it relates to adolescents. Curr Psychiatry Rep. 2019;21(9):93. 10.1007/s11920-019-1075-3.

[179] Gradus JL, Rosellini AJ, Horvath-Puho E, Jiang T, Street AE, Galatzer-Levy I, et al. Predicting sex-specific non-fatal suicide attempt risk using machine learning and data from Danish national registries. Am J Epidemiol. 2021;190(12):2517-2527.

[180] Just MA, Pan L, Cherkassky VL, McMakin DL, Cha C, Nock MK, et al. Machine learning of neural representations of suicide and emotion concepts identifies suicidal youth. Nat Hum Behav. 2017;1:911-919. 10.1038/s41562-017-0234-y.

[181] Dukart J, Weis S, Genon S, Eickhoff SB (2021). Towards increasing the clinical applicability of machine learning biomarkers in psychiatry. Nat Hum Behav.

[182] Bossarte RM, Kennedy CJ, Luedtke A, Nock MK, Smoller JW, Stokes C, et al. Invited commentary: New directions in machine learning analyses of administrative data to prevent suicide-related behaviors. Am J Epidemiol. 2021;190(12):2528-2533.

