# Supplementary material for: Peripheral and neural correlates of self-harm in children and adolescents: a scoping review
Source: BMC Psychiatry. 2022 May 4;22:318. doi: 10.1186/s12888-022-03724-6 (PMC9066835; doi:10.1186/s12888-022-03724-6)
Supplement: Supplementary file 2 — Additional file 2. Search Strategy: PubMed & Embase, January 1, 1980-May 6, 2020. [file 12888_2022_3724_MOESM2_ESM.docx]

**Supplement 2 Search Strategy: PubMed & Embase, January 1, 1980-May 6, 2020**

| **Query #** | **PubMed** |
| --- | --- |
| 1 | child: ("child"[MeSH Terms] OR "child"[All Fields] OR "children"[All Fields] OR "child s"[All Fields] OR "children s"[All Fields] OR "childrens"[All Fields] OR "childs"[All Fields]) AND "humans"[MeSH Terms] |
| 2 | **adolescent:** ("adolescences"[All Fields] OR "adolescency"[All Fields] OR "adolescent"[MeSH Terms] OR "adolescent"[All Fields] OR "adolescence"[All Fields] OR "adolescents"[All Fields] OR "adolescent s"[All Fields]) AND "humans"[MeSH Terms] |
| 3 | **pediatric:** ("paediatrics"[All Fields] OR "pediatrics"[MeSH Terms] OR "pediatrics"[All Fields] OR "paediatric"[All Fields] OR "pediatric"[All Fields]) AND "humans"[MeSH Terms] |
| 4 | **1 OR 2 OR 3** |
| 5 | self-injurious behavior: ("self injurious behaviour"[All Fields] OR "self injurious behavior"[MeSH Terms] OR ("self injurious"[All Fields] AND "behavior"[All Fields]) OR "self injurious behavior"[All Fields] OR ("self"[All Fields] AND "injurious"[All Fields] AND "behavior"[All Fields]) OR "self injurious behavior"[All Fields]) AND "humans"[MeSH Terms] |
| 6 | **suicide:** ("suicid"[All Fields] OR "suicidal"[All Fields] OR "suicidality"[All Fields] OR "suicidally"[All Fields] OR "suicidals"[All Fields] OR "suicide"[MeSH Terms] OR "suicide"[All Fields] OR "suicides"[All Fields] OR "suicide s"[All Fields] OR "suicided"[All Fields] OR "suiciders"[All Fields]) AND "humans"[MeSH Terms] |
| 7 | **5 OR 6** |
| 8 | **4 AND 7** |
| 9 | **biological correlate:** "biological products"[MeSH Terms] OR ("biological"[All Fields] AND "products"[All Fields]) OR "biological products"[All Fields] OR "biologicals"[All Fields] OR "biological factors"[MeSH Terms] OR ("biological"[All Fields] AND "factors"[All Fields]) OR "biological factors"[All Fields] OR "biologics"[All Fields] OR "biologically"[All Fields] OR "biology"[MeSH Terms] OR "biology"[All Fields] OR "biologic"[All Fields] OR "biological"[All Fields]; "correlate"[All Fields] OR "correlated"[All Fields] OR "correlates"[All Fields] OR "correlating"[All Fields] OR "correlation"[All Fields] OR "correlation's"[All Fields] OR "correlations"[All Fields] OR "correlative"[All Fields] OR "correlatives"[All Fields] |
| 10 | evoked potentials: "evoked potentials"[MeSH Terms] OR ("evoked"[All Fields] AND "potentials"[All Fields]) OR "evoked potentials"[All Fields] |
| 11 | cortisol: "cortisol's"[All Fields] OR "cortisole"[All Fields] OR "hydrocortisone"[MeSH Terms] OR "hydrocortisone"[All Fields] OR "cortisol"[All Fields] OR "cortisols"[All Fields] |
| 12 | autonomic nervous system: "autonomic nervous system"[MeSH Terms] OR ("autonomic"[All Fields] AND "nervous"[All Fields] AND "system"[All Fields]) OR "autonomic nervous system"[All Fields] |
| 13 | **pain:** "pain"[MeSH Terms] OR "pain"[All Fields] |
| 14 | nutrition: "nutrition's"[All Fields] OR "nutritional status"[MeSH Terms] OR ("nutritional"[All Fields] AND "status"[All Fields]) OR "nutritional status"[All Fields] OR "nutrition"[All Fields] OR "nutritional sciences"[MeSH Terms] OR ("nutritional"[All Fields] AND "sciences"[All Fields]) OR "nutritional sciences"[All Fields] OR "nutritional"[All Fields] OR "nutritionals"[All Fields] OR "nutritions"[All Fields] OR "nutritive"[All Fields] |
| 15 | lipids: "lipid's"[All Fields] OR "lipidate"[All Fields] OR "lipidated"[All Fields] OR "lipidates"[All Fields] OR "lipidation"[All Fields] OR "lipidations"[All Fields] OR "lipide"[All Fields] OR "lipides"[All Fields] OR "lipidic"[All Fields] OR "lipids"[MeSH Terms] OR "lipids"[All Fields] OR "lipid"[All Fields] |
| 16 | inflammation: "inflammation"[MeSH Terms] OR "inflammation"[All Fields] OR "inflammations"[All Fields] OR "inflammation's"[All Fields] |
| 17 | cytokines: "cytokin"[All Fields] OR "cytokine's"[All Fields] OR "cytokines"[MeSH Terms] OR "cytokines"[All Fields] OR "cytokine"[All Fields] OR "cytokinic"[All Fields] OR "cytokins"[All Fields] |
| 18 | neuroimaging: "neuroimage"[All Fields] OR "neuroimaged"[All Fields] OR "neuroimagers"[All Fields] OR "neuroimages"[All Fields] OR "neuroimaging"[MeSH Terms] OR "neuroimaging"[All Fields] OR "neuroimagings"[All Fields] |
| 19 | neurotransmitter: "neurotransmitter agents"[Pharmacological Action] OR "neurotransmitter agents"[MeSH Terms] OR ("neurotransmitter"[All Fields] AND "agents"[All Fields]) OR "neurotransmitter agents"[All Fields] OR "neurotransmitter"[All Fields] OR "neurotransmitters"[All Fields] |
| 20 | gamma aminobutyric acid: "gamma-aminobutyric acid"[MeSH Terms] OR ("gamma-aminobutyric"[All Fields] AND "acid"[All Fields]) OR "gamma-aminobutyric acid"[All Fields] OR ("gamma"[All Fields] AND "aminobutyric"[All Fields] AND "acid"[All Fields]) OR "gamma aminobutyric acid"[All Fields] |
| 21 | opioids: "analgesics, opioid"[Pharmacological Action] OR "analgesics, opioid"[MeSH Terms] OR ("analgesics"[All Fields] AND "opioid"[All Fields]) OR "opioid analgesics"[All Fields] OR "opioid"[All Fields] OR "opioids"[All Fields] OR "opioid's"[All Fields] |
| 22 | electroencephalography: "electroencephalographies"[All Fields] OR "electroencephalography"[MeSH Terms] OR "electroencephalography"[All Fields] |
| 23 | sleep: "sleep"[MeSH Terms] OR "sleep"[All Fields] OR "sleeping"[All Fields] OR "sleeps"[All Fields] OR "sleep's"[All Fields] (201,867) |
| 24 | neurotropic factor all fields |
| 25 | S-100B all fields |
| 26 | estrogens: "estrogen's"[All Fields] OR "estrogene"[All Fields] OR "estrogenes"[All Fields] OR "estrogenic"[All Fields] OR "estrogenically"[All Fields] OR "estrogenicities"[All Fields] OR "estrogenicity"[All Fields] OR "estrogenization"[All Fields] OR "estrogenized"[All Fields] OR "oestrogen"[All Fields] OR "estrogens"[Pharmacological Action] OR "estrogens"[MeSH Terms] OR "estrogens"[All Fields] OR "estrogen"[All Fields] OR "oestrogen's"[All Fields] OR "oestrogenic"[All Fields] OR "oestrogenically"[All Fields] OR "oestrogenicity"[All Fields] OR "oestrogenization"[All Fields] OR "oestrogens"[All Fields] |
| 27 | testosterone: "testosterone"[MeSH Terms] OR "testosterone"[All Fields] OR "testosteron"[All Fields] OR "testosterones"[All Fields] OR "testosterone's"[All Fields] |
| 28 | growth hormone: "growth hormone"[MeSH Terms] OR ("growth"[All Fields] AND "hormone"[All Fields]) OR "growth hormone"[All Fields] |
| 29 | 9 OR 10 OR 11 OR 11 OR 13 OR 14 OR 15 OR 16 OR 17 OR 18 OR 19 OR 20 OR 21 OR 22 OR 23 OR 24 OR 25 OR 26 OR 27 OR 28 |
| 30 | 8 AND 29 |
| **Query #** | **Embase** |
| 1 | (child or adolescent or pediatric).mp. [mp=title, abstract, heading word, drug trade name, original title, device manufacturer, drug manufacturer, device trade name, keyword, floating subheading word, candidate term word] |
| 2 | limit 1 to (human and english language and yr="1980 - 2020") |
| 3 | (self-mutilation or suicide).mp. [mp=title, abstract, heading word, drug trade name, original title, device manufacturer, drug manufacturer, device trade name, keyword, floating subheading word, candidate term word] |
| 4 | limit 3 to (human and english language and yr="1980 - 2020") |
| 5 | 2 and 4 |
| 6 | biological correlate.mp. |
| 7 | limit 6 to (human and english language and yr="1980 - 2020") |
| 8 | evoked potential.mp. or evoked response/ |
| 9 | limit 8 to (human and english language and yr="1980 - 2020") |
| 10 | cortisol.mp. or hydrocortisone/ |
| 11 | limit 10 to (human and english language and yr="1980 - 2020") |
| 12 | autonomic nervous system.mp. or exp autonomic nervous system/ |
| 13 | limit 12 to (human and english language and yr="1980 - 2020") |
| 14 | *pain/ or pain.mp. |
| 15 | limit 14 to (human and english language and yr="1980 - 2020") |
| 16 | child nutrition/ or adolescent nutrition/ or nutrition.mp. or infant nutrition/ or nutrition/ |
| 17 | limit 16 to (human and english language and yr="1980 - 2020") |
| 18 | lipid.mp. or lipid blood level/ or lipid level/ or lipid/ or lipid diet/ |
| 19 | limit 18 to (human and english language and yr="1980 - 2020") |
| 20 | cardiovascular inflammation/ or chronic inflammation/ or inflammation.mp. or inflammation/ or nervous system inflammation/ |
| 21 | limit 20 to (human and english language and yr="1980 - 2020") |
| 22 | interleukin 6/ or cytokine/ or cytokin*.mp. |
| 23 | limit 22 to (human and english language and yr="1980 - 2020") |
| 24 | exp neuroimaging/ or functional neuroimaging/ or neuroimaging.mp. |
| 25 | limit 24 to (human and english language and yr="1980 - 2020") |
| 26 | neurotransmitter receptor/ or neurotransmitter uptake/ or neurotransmitter/ or neurotransmitter transporter/ or neurotransmitter release/ or neurotransmitter*.mp. |
| 27 | limit 26 to (human and english language and yr="1980 - 2020") |
| 28 | gamma aminobutyric acid.mp. or 4 aminobutyric acid/ |
| 29 | limit 28 to (human and english language and yr="1980 - 2020") |
| 30 | opioid*.mp. |
| 31 | limit 30 to (human and english language and yr="1980 - 2020") (84778) |
| 32 | electroencephalography.mp. or electroencephalography/ (135527) |
| 33 | limit 32 to (human and english language and yr="1980 - 2020") (67901) |
| 34 | sleep arousal disorder/ or sleep pattern/ or REM sleep/ or sleep waking cycle/ or fragmented sleep/ or sleep hygiene/ or sleep quality/ or circadian rhythm sleep disorder/ or central sleep apnea syndrome/ or stage 2 sleep/ or stage 1 sleep/ or sleep time/ or slow wave sleep/ or REM sleep latency/ or sleep.mp. or sleep deprivation/ or sleep disorder/ or REM sleep deprivation/ or night sleep/ or sleep stage/ or sleep/ or nonREM sleep/ |
| 35 | limit 34 to (human and english language and yr="1980 - 2020") |
| 36 | estradiol plus progesterone plus testosterone/ or testosterone blood level/ or testosterone/ or testosterone.mp. or testosterone 17beta dehydrogenase/ or testosterone metabolism/ |
| 37 | limit 36 to (human and english language and yr="1980 - 2020") |
| 38 | estrogen release/ or estrogen.mp. or estrogen deficiency/ or estrogen activity/ or estrogen blood level/ or estrogen metabolism/ or estrogen/ or estrogen receptor/ |
| 39 | limit 38 to (human and english language and yr="1980 - 2020") |
| 40 | growth hormone.mp. or growth hormone/ |
| 41 | limit 40 to (human and english language and yr="1980 - 2020") (54956) |
| 42 | protein S100B/ or S-100B.mp. |
| 43 | limit 42 to (human and english language and yr="1980 - 2020") |
| 44 | brain derived neurotrophic factor/ or brain-derived neurotropic factor.mp. |
| 45 | limit 44 to (human and english language and yr="1980 - 2020") |
| 46 | 7 or 9 or 11 or 13 or 15 or 17 or 19 or 21 or 23 or 25 or 27 or 29 or 31 or 33 or 35 or 37 or 39 or 41 or 43 or 45 |
| 47 | 5 and 46 |
