# Supplementary material for: Peripheral and neural correlates of self-harm in children and adolescents: a scoping review
Source: BMC Psychiatry. 2022 May 4;22:318. doi: 10.1186/s12888-022-03724-6 (PMC9066835; doi:10.1186/s12888-022-03724-6)
Supplement: Supplementary file 3 — Additional file 3. Study details for peripheral correlates for self-harm in children and adolescents. [file 12888_2022_3724_MOESM3_ESM.docx]

**Supplement 3** **Study details of peripheral correlates of self-harm in children and adolescents**

| **Author, Date**  **Country** | **Self-Harm**  **Type** | **Study Design** | **-N**  **-Ages**  **-Source**  **-Diagnosis**  **-Controls** | **% Girls** | **% White** | **Self-Harm Data Source** | **-Correlate**  **-Measure** | **Main Findings** | **Bias Rating** |
| --- | --- | --- | --- | --- | --- | --- | --- | --- | --- |
| ***Stress Response System*** | | | | | | | | | |
| *Hypothalamic Pituitary Adrenal Axis (HPA Axis)* | | | | | | | | | |
| Robbins, Alessi 1985 US [54] | Suicidality | Case-Control | -N = 45  -13-18 yrs  -Inpt  -None  -Psychiatric controls | 52% | Not given | Diagnostic interview, SADS | -Circadian rhythm  -DST  -Cortisol | -Associated with cortisol  non-suppression | Fair |
| Rosenthal et al. 1986 US [55] | Suicidality | Case-Control | -N = 41  -3-5 yrs  -Inpt  -None  -Psychiatric controls | 11% | Not given | -Clinical records  -Clinician rating scale or interview, PDS | -Circadian rhythm  -DST  -Cortisol | -Not associated with cortisol non-suppression | Poor |
| Dahl et al. 1991a US [56] | Suicidality | Case-Control | -N = 59  -12-18 yrs  -Inpt, Outpt, Cmty  -MDD  -Healthy controls | 61% | 85% | Diagnostic interview,  K-SADS | -Circadian rhythm  -Cortisol levels /24 hours | -Associated with later  cortisol nocturnal nadir | Good |
| Dahl et al. 1992a US [57] | Suicidality | Case-Control | -N = 61  -14-18 yrs  -Inpt, outpt,  cmty  -MDD  -Healthy controls | 59% | 85% | Diagnostic interview,  K-SADS | -Circadian rhythm  -DST  -Cortisol | -Not associated  cortisol non-suppression | Good |
| Ghaziuddin et al. 2014 US [58] | Suicidality | Case-Control | -N = 44  -13-17 yrs  -Inpt  -MDD  -Psychiatric controls | 66% | 84% | Clinician rating scale or interview, SSBS | -Reactivity  -mCPP challenge  -Cortisol | -Associated different pattern cortisol secretion males with suicidality | Good |
| Young et al. 2010  Scotland [59] | Suicidality | Cross-Sectional | -N = 501  -15 yrs  -Cmty  -None | 80% | Not given | Diagnostic interview, DISC | -Circadian rhythm  -9AM cortisol | -Not associated with  cortisol levels | Good |
| Pfeffer et al. 1991 US [60] | Suicidality | Cohort | -N = 49  -6-12 yrs  -Inpt  -None | 29% | 59% | Clinician rating scale or interview, SSBS | -Circadian rhythm  -DST admission, 7 weeks later  -Cortisol | -Not associated  with cortisol non-suppression, but higher 4 PM pre-DST level predicted suicidality 7 weeks | Good |
| Giletta, et al. 2015 US [61] | Suicidality | Cohort | -N = 138  -12-16 yrs  -Inpt, cmty  -MH concerns | 100% | 67% | Clinician rating scale or interview, SITBI | -Reactivity  -TSST, baseline  -Cortisol | -Baseline  hyperreactivity associated with ideation, 3-mo persistence | Fair |
| Eisenlohr-Moul et al. 2018 US [62] | Suicidality | Cohort | -N = 220  -12-16 yrs  -Inpt, outpt, cmty  -MH concerns | 100% | 64% | Clinician rating scale or interview, SITBI | -Reactivity  -TSST baseline  -Cortisol | -Baseline hyporeactivity associated with behaviors, 18-mo persistence | Fair |
| Reichl et al, 2016 Germany [63] | NSSI | Case-Control | - N = 52  -14-18 yrs  -Inpt,outpt, cmty  -None  -Healthy controls | 92% | Not given | Clinician rating scale or interview, SITBI | -Circadian rhythm  -3 days cortisol secretion, hair levels  -Cortisol | -Associated higher total secretion, higher CAR  -Not associated hair cortisol | Good |
| Klimes-Dougan et al. 2019 US [64] | NSSI | Case-Control | -N = 162  -12-19 yrs  -Inpt, outpt, cmty  -MDD  -Healthy controls | 67% | 72% | Diagnostic interview,  K-SADS | -Reactivity  -TSST  -Cortisol | -Associated with hyporeactivity | Fair |
| Reichl, et al. 2019 Germany [65] | NSSI | Case-Control | -N = 64  -12-19 yrs  -Inpt, outpt  -None  -Sibling controls | 86% | Not given | Clinician rating scale or interview, SITBI | -Reactivity, Circadian rhythm  -Response to adverse events recall  -Hair levels  -Cortisol | -Associated with larger pre-post decrease to recalling events  -Higher hair levels | Good |
| Beauchaine  et al. 2015 US [66] | Any self-harm | Case-Control | -N = 57  -13-17 yrs  -Inpt, outpt, cmty  -MDD  -Psychiatric controls | 100% | Not given | -Clinician rating scale or interview, L-SASI  -Self-report, SIQ | -Circadian rhythm  -DST  -Cortisol | -Ideation associated negatively with cortisol suppression,  -L-SASI score not associated | Good |
| Plener et al. 2016  Germany [67] | Any self-harm | Cohort | -N = 130  -15-19 yrs  -Cmty  -None | 100% | Not given | Self-report, YSR, YASR | -Reactivity  -TSST  -Cortisol | -Associated with hyporeactivity | Good |
| *Autonomic Nervous System (ANS)* | | | | | | | | | |
| Yang et al., 2019 Hungary [68] | Suicidality | Case-Control | -N = 399  -11-19 yrs  -Outpt, cmty  -MDD  -Healthy controls, Psychiatric controls | 35% | Not given | Diagnostic interview, ISCA-D | -Reactivity  -Video challenge, unsolvable puzzle  - RSA, PEP | -Not associated response to sad video  -Associated with less parasympathetic and greater sympathetic response to puzzle, but only vs. psychiatric controls, not healthy controls | Good |
| Giletta et al., 2017 US [69] | Suicidality | Cohort | -N=132  -12-17 yrs  -Inpt, cmty  -MH concerns | 100% | 68% | Clinician rating scale or interview, SITBI | -Reactivity  -TSST  -RSA baseline | -Greater parasympathetic withdrawal TSST associated with more ideation over 9- month period | Good |
| Koenig et al. 2017a Germany [70] | NSSI | Case-Control | -N = 60  -12-17 yrs  -Outpt, cmty  -None  -Healthy controls | 100% | Not given | Clinician rating scale or interview, SITBI | -Resting state  -HR, HRV | -Not associated with HR, HRV | Fair |
| Crowell et al. 2005 US [51] | Any self-harm | Case-control | -N = 46  -14-18 yrs  -Inpt, outpt, cmty  -None  -Healthy controls | 100% | 74% | Clinician rating scale or interview, LPC | -Resting, Reactivity  -Video challenge  -RSA, PEP, EDR | -Associated lower baseline  parasympathetic tone  -Associated greater vagal reactivity  -Not associated  EDR | Fair |
| Crowell et al. 2012 US [71] | Any self-harm | Case-control | -N = 75  -13-17 yrs  -Inpt, outpt  -MDD  -Healthy controls, Psychiatric controls | 100% | 70% | Clinician rating scale or interview, L-SASI | -Resting state and reactivity  -Video challenge  -EDR | -Associated with lower  EDR resting state  -Not associated with reactivity | Good |
| Wielgus et al. 2016 US [72] | Any self-harm | Cohort | -N = 108  -11-14 yrs  -Cmty  -None | 54% | 71% | -Diagnostic interview, K-SADS  -Self-report, YSR, CDI, CBCL | -Resting, Reactivity at baseline  -Cognitive challenge  -RSA | -6-month self-harm not associated parasympathetic tone or reactivity, but slower recovery from stressor | Fair |
| Aldrich et al. 2018 US [73] | Any self-harm | Cohort | -N = 121  -10-14 yrs  -Cmty  -None | 55% | Not given | -Diagnostic interview, K-SADS  -Self-report, YSR, CDI, CBCL | -Reactivity at baseline  - Cognitive challenge  -EDR | -6-month self-harm not associated sympathetic reactivity  -6-month self-harm associated with lower reactivity in high impulsivity sub-group | Poor |
| *HPA Axis and ANS* | | | | | | | | | |
| Kaess et al. 2012 Germany [74] | NSSI | Case-control | -N = 28  -14-18 yrs  -Outpt, cmty  -None  -Healthy controls | 100% | Not given | Self-report, FASM | -Reactivity HPA axis, ANS  -TSST  -Cortisol, HR | -Associated cortisol hyporeactivity  -Not associated with HR | Fair |
| Koenig et al. 2017b Germany [75] | NSSI | Case-control | -N = 60  -12-17 yrs  -Outpt, cmty  -None  -Healthy controls | 100% | Not given | Clinician rating scale or interview, SITBI | -Reactivity HPA axis, ANS  -CPT  -Cortisol, HRV, BP, HR | -Associated cortisol  hyperreactivity  - Not associated with HRV, BP, HR reactivity  -Associated with less parasympathetic arousal pre-CPT and longer recovery | Fair |
| ***Serotonin System*** | | | | | | | | | |
| Modai et al. 1989 Israel [76] | Suicidality | Case-control | -N = 34  -“adolescents”  -Inpt  -None  -Psychiatric controls | Not given | Not given | Diagnostic interview,  K-SADS | -Platelet 5-HT function  -Labelled 5-HT uptake | -Not associated with uptake | Good |
| Ambrosini et al. 1992 US [77] | Suicidality | Case-control | -N = 32  -8-17 yrs  -Inpt, outpt  -MDD  -Psychiatric controls | 56% | Not given | Diagnostic interview,  K-SADS | -Platelet 5-HT function  **-**IMI binding sites | -Associated with fewer sites in MDD + suicidality subjects | Poor |
| Pfeffer et al. 1998 US [78] | Suicidality | Case-control | -N = 110  -6-12 yrs  -Inpt, cmty  -None  -Healthy controls | 45% | 59% | Clinician rating scale or interview, SABS | -Peripheral serotonin, tryptophan levels  -Whole blood 5-HT, whole blood tryptophan,  -5-HT- amplified platelet aggregation | -Associated with lower tryptophan level in attempts  -Not associated with whole blood 5-HT or platelet aggregation | Fair |
| Tyano et al. 2006 Israel [79] | Suicidality | Case-control | -N = 211  -13-19 yrs  -Inpt, cmty  -None  -Healthy controls | 59% | Not given | Clinician rating scale or interview, SPI | -Peripheral serotonin levels  -Plasma 5-HT | -Higher 5-HT levels associated with all patients vs. healthy controls  -Positive correlation 5-HT and SPI scores in suicidal inpatients | Good |
| Pine et al 1995  US [80] | Suicidality | Cohort | -N = 121  -10-18  -Inpt, outpt, previous study  -None  -Psychiatric controls | 66% | Not given | Self-report, BSS | -Platelet 5-HT function  -IMI binding | -Not associated with number binding sites  -Associated with seasonal variation in number of sites | Fair |
| Clark et al. 2003 US [81] | Suicidality | Cohort | -N = 60  -14-17 yrs  -Inpt ,outpt, comty  -AUD  -Healthy controls | 55% | 85% | -Diagnostic interview, K-SADS  -Self-report, BSS | -Peripheral tryptophan levels predicting suicidality  -Serum levels and ratio tryptophan to other amino acids | - Serum levels or ratio not associated at baseline  -Low baseline ratio predicted 5-yr suicidality in AUD/MDD | Fair |
| Crowell et al. 2005 US [51] | Any self-harm | Case-control | -N = 46  -14-18 yrs  -Inpt, outpt, cmty  -None  -Healthy controls | 100% | 74% | Clinician rating scale or interview, LPC | -Peripheral 5-HT  -Whole blood 5-HT | -Associated with lower whole blood 5-HT | Fair |
| Crowell et al. 2008 US [82] | Any self-harm | Case-control | -N = 41  -14-18 yrs  -Inpt, outpt, cmty  -None  -Healthy controls | 91% | 78% | Clinician rating scale or interview, L-SASI | -Peripheral 5-HT  -Whole blood 5-HT | -Associated with lower whole blood 5-HT | Good |
| ***Sleep*** | | | | | | | | | |
| Dahl et al, 1990  US [83] | Suicidality | Case-control | -N = 59  -12-17 yrs  -Outpt, cmty  -MDD  -Healthy controls | 58% | 91% | Diagnostic interview,  K-SADS | -Sleep characteristics  -PSG X 3 nights | -Associated with longer sleep latency | Fair |
| Dahl et al. 1991b US [84] | Suicidality | Case-control | -N = 54  -6-11 yrs  -Inpt, outpt, cmty  -MDD  -Healthy controls | 33% | 81% | Diagnostic interview,  K-SADS | -Sleep characteristics  -PSG X 2 nights | -Not associated any sleep characteristic | Good |
| Emslie et al. 1994 US [85] | Suicidality | Case-control | -N = 48  -13-17 yrs  -Inpt, cmty  -MDD  -Healthy controls | 68% | 87% | Diagnostic interview, DICA | -Sleep characteristics  -PSG X 3 nights | -Not associated with any sleep characteristic | Fair |
| McCracken et al. 1997 US [86] | Suicidality | Case-control | -N = 33  -12-18 yrs  -Outpt, cmty  -MDD  -Healthy controls | 58% | Not given | Clinician rating scale or interview, HDRS | -Sleep characteristics  -Sleep in response to scopolamine challenge  -PSG X 4 nights | -Associated with longer sleep duration, shorter percentage of Stage 3 sleep, shorter total delta sleep  -Cholinergic challenge associated with more REM transitions | Fair |
| Boafo et al. 2018 Canada [87] | Suicidality | Case-control | -N = 34  -12-17 yrs  -Inpt, research database  -MDD  -Healthy controls | 82% | Not given | Self-report instrument, SBQ-R | -Sleep characteristics  -PSG X 2 nights | -Associated with longer sleep and REM latency, higher percentage of NREM1, higher REM density | Good |
| Singareddy et al. 2013 US [88] | Suicidality | Cohort | -N = 693  -5-12 yrs  -Cmty  -None | 53% | Not given | Self-report instrument. PBS | -Sleep characteristics  -PSG X 1 night | -Associated with higher percentage REM sleep | Good |
| ***Neuromodulators*** | | | | | | | | | |
| Bilgiç et al. 2020 Turkey [89] | Suicidality | Case-Control | -N = 110  -11-19 yrs  -Outpt, cmty  -MDD  -Healthy controls | 76% | Not given | Clinician rating scale or interview, CSSRS | -Neurotrophin levels  -Fasting serum BDNF, GDNF, NGF, NTF3 | -Not associated with any neurotrophin level | Good |
| Falcone et al. 2010 US [52] | Suicidality | Case-Control | -N = 84  -12-18 yrs  -Inpt, cmty  -Psychosis, mood disorders  -Healthy controls, Psychiatric controls | 45% | 61% | Clinician rating scale or interview, BPRS-C | -Peripheral S100B levels  -Serum levels | -Associated with higher S100B levels | Good |
| Falcone et al. 2015 US [90] | Suicidality | Case-Control | -N = 115  -7-18 yrs  -Inpt, cmty  - Psychosis, mood disorders  -Healthy controls, Psychiatric controls | 47% | 60% | Clinician rating scale or interview, BPRS-C | -Peripheral S100B levels  -Serum | -Associated with higher S100B levels | Good |
| Kavurma et al. 2017 Turkey [91] | Any self-harm | Case-Control | -N = 105  -12-18 yrs  -Outpt, cmty  -None  -Healthy controls | 73% | Not given | Diagnostic interview,  K-SADS  Self-report instrument, ISAS | -BDNF levels  -Fasting serum | -Not associated with BDNF levels in NSSI or suicidality (analyzed separately) | Good |
| ***Immune System*** | | | | | | | | | |
| Gabbay et al. 2009 US [92] | Suicidality | Case-Control | -N = 45  -12-19 yrs  -Inpt, outpt, cmty  -MDD  -Healthy controls, Psychiatric controls | 60% | Not given | Self-report instrument, BSS | -Cytokine levels  -Fasting plasma IFN-$\gamma$, TNFα, IL-6, IL-1β, and IL-4 levels | -Associated with decreased TNFα, increased IFN-$\gamma$ | Good |
| Falcone et al. 2010  US *(supplement data)* [52] | Suicidality | Case-Control | -N = 84  -12-18 yrs  -Inpt, cmty  -Psychosis, mood disorders  -Healthy controls, Psychiatric controls | 45% | 61% | Clinician rating scale or interview, BPRS-C | -Cytokine levels  -Serum IL-1α, IL-1ß, IL-2, IL-4, IL-6, IL-8, IL-10, IFN-$\gamma$,TNFα | -High suicidality score associated with increased IL-ß, IL-8 | Fair |
| Amitai et al. 2019 Israel [93] | Suicidality | Non-Controlled Pre-Post Intervention | -N = 95  -11-16 yrs  -Outpt  -MDD or anxiety disorders | 62% | Not given | -Self-report instruments, SIQ  -Clinician rating scale or interview, CSSRS, CDRS | -Cytokine levels in response FLX  -Plasma levels TNFα, IL-6,  IL-1β pre-post FLX treatment | -Post-FLX suicidality associated with greater increase IL-6 | Fair |
| ***Lipid Levels*** | | | | | | | | | |
| Glueck et al. 1994 US [94] | Suicidality | Case-Control | -N = 1268  -5-18 yrs  -Inpt, previous research studies  -None  -Healthy controls | 37% | 44% | Clinical records | -Plasma lipid levels  -Fasting cholesterol and triglycerides | -Associated with lower cholesterol  -Not associated with triglycerides | Good |
| Plana et al. 2010 Spain [95] | Suicidality | Case-Control | -N = 120  -8-18 yrs  -Inpt  -None  -Psychiatric controls | 67% | Not given | Clinical records | -Plasma lipid levels  -Fasting cholesterol | -Associated with lower cholesterol | Good |
| ***Pituitary Hormones*** | | | | | | | | | |
| Ryan et al. 1988 US [96] | Suicidality | Case-Control | -N = 43  -12-17 yrs  -Inpt, outpt, cmty  -MDD  -Healthy controls | 42% | 65% | Diagnostic interview,  K-SADS | -GH reactivity  -DMI challenge | -Associated with lower post-DMI levels | Poor |
| Dahl et al.1992b US [97] | Suicidality | Case-Control | -N = 88  -12-18 yrs  -Inpt, outpt, cmty  -MDD  -Healthy controls | 46% | 57% | Diagnostic interview,  K-SADS | -GH 24-hour rhythm  -Serum GH X 24 hours | -Associated lower GH secretion first 4 hours sleep  -Associated with lower total GH secretion during sleep | Fair |

5-HT - 5-hydroxytryptamine (serotonin), ANS - Autonomic Nervous System, AUD - Alcohol Use Disorder, BDNF - Brain-Derived Neurotrophin Factor, BP - Blood Pressure, BPRS-C - Brief Psychiatric Rating Scale-Children, BSS - Beck Scale for Suicide Ideation, CAR - Cortisol Awakening Response, CBCL - Child Behavior Checklist, CDI - Child Depression Inventory, CDRS - Child Depression Rating Scale, Cmty – Community, CPT - Cold Pressor Test, CSSRS -Columbia Suicide Severity Rating Scale, DICA - Diagnostic Interview for Children and Adolescents, DISC - Diagnostic Interview Schedule for Children, DMI - Desipramine, DST -Dexamethasone Suppression Test, EDR - Electrodermal response, FASM - Functional Assessment of Self-Mutilation, FLX – Fluoxetine, GDNF - Glial-Derived Neurotrophin Factor, GH - Growth Hormone, HDRS - Hamilton Depression Rating Scale, HPA - Hypothalamic Pituitary Adrenal, HR - Heart Rate, HRV - Heart Rate Variability, IFN-$\gamma$ - Interferon-Gamma, IL-10 - Interleukin 10, IL-1α - Interleukin 1-Alpha, IL-1β - Interleukin 1-Beta, IL-2 - Interleukin 2, IL-4 - interleukin 4, IL-6 - Interleukin 6, IL-8 - Interleukin 8, IMI – Imipramine, Inpt -Inpatients, ISAS - Inventory of Statements about Self-Injury, ISCA-D - Interview Schedule for Children and Adolescents-Diagnostic version, K-SADS - Kiddie-Schedule for Affective Disorders and Schizophrenia, LPC - Lifetime Parasuicide Count, L-SASI - Lifetime Suicide Attempt Self-Injury Interview, mCPP - m-Chlorophenylpiperazine, MDD - Major Depressive Disorder, MH - Mental Health, NGF - Nerve Growth Factor, NREM1 – Non-REM, Stage 1, NSSI - Non Suicidal Self-Injury, NTF3 - Neurotrophin-3 Factor, Outpt - Outpatients, PBS - Pediatric Behavior Scale, PDS - Preschool Depression Scale, PEP - Cardiac Pre-Ejection Period, PSG – polysomnography, REM - Rapid Eye Movement, RSA - Respiratory Sinus Arrhythmia, S100B - S100 - calcium-binding protein B, SABS - Suicidal and Assaultive Behavior Scales, SADS - Schedule for Affective Disorders and Schizophrenia, SBQ-R - Suicide Behaviors Questionnaire-Revised, SIQ - Suicidal Ideation Questionnaire, SITBI - Self-Injurious Thoughts and Behaviors Interview, SPI - Suicide Potential Inventory, SSBS - Spectrum of Suicidal Behaviors Scale, TNFα - Tumour Necrosing Factor-Alpha, TSST - Trier Social Stress Test, YASR - Young Adult Self-Report, Yrs - years, YSR - Youth Self-Report
