# Supplementary material for: Peripheral and neural correlates of self-harm in children and adolescents: a scoping review
Source: BMC Psychiatry. 2022 May 4;22:318. doi: 10.1186/s12888-022-03724-6 (PMC9066835; doi:10.1186/s12888-022-03724-6)
Supplement: Supplementary file 4 — Additional file 4. Study details for neural correlates of self-harm in children and adolescents. [file 12888_2022_3724_MOESM4_ESM.docx]

**Supplement 4** **Study details for neural correlates of self-harm in children and adolescents**

| **Author, Date**  **Country** | **Self-Harm**  **Type** | **Study Design** | **-N**  **-Ages**  **-Source**  **-Diagnosis**  **-Controls** | **% Girls** | **% White** | **Self-Harm Data Source** | **-Correlate**  **-Measure** | **Main Findings** | **Bias Rating** |
| --- | --- | --- | --- | --- | --- | --- | --- | --- | --- |
| ***Brain Function, Imaging*** | | | | | | | | | |
| *Brain Reactivity* | | | | | | | | | |
| Pan et al. 2011  US [98] | Suicidality | Case-Control | -N = 44  -13-17 yrs  -Previous studies, registry, cmty  -MDD  -Healthy controls, Psychiatric controls | 57% | Not given | -Clinician rating scale or interview, CSSRS  -Self-report, SIQ, BSIS | -Activity during response inhibition  -fMRI, Go-No Go Task | -Not associated with abnormal activity in response inhibition circuitry | Fair |
| Pan et al. 2013a US [53] | Suicidality | Case-Control | -N = 44  -13-17 yrs  -Previous studies, registry, cmty  -MDD  -Healthy controls, Psychiatric controls | 57% | Not given | -Clinician rating scale or interview, CSSRS  -Self-report, SIQ, BSIS | -Activity during facial emotion processing  -fMRI, FE  EST | -Associated greater activation in rdACG, bilateral primary sensory cortices, dLPFC, rMTG for angry faces  -Associated with less activation in visual, sensory, PF cortices, ACG for happy faces | Fair |
| Pan et al. 2013b US [99] | Suicidality | Case-Control | -N = 42  -12-17 yrs  -Previous studies, registry, cmty  -MDD  -Healthy controls, Psychiatric controls | 55% | Not given | -Clinician rating scale or interview, CSSRS  -Self-report, SIQ, BSIS | -Activation during decision-making task  -fMRI, IGT | -Not associated with difference from healthy controls | Fair |
| Quevedo et al. 2016a US [100] | Suicidality | Case-Control | -N = 119  -11-18 yrs  -Inpt, outpt, cmty  -MDD  -Healthy controls, Psychiatric controls | 61% | 62% | -Diagnostic interview, K-SADS  -Clinician rating scale or interview, CDRS | -Activation during self-identity processing  -fMRI,  ESOM-Q | -Associated with increased activation bilateral cuneus, MOG across all conditions  -Associated with hypoactivity mPFC to self faces and higher mPFC activity to other faces after adjusting for MDD | Fair |
| Harms et al. 2019 US [101] | Suicidality | Case-Control | -N = 126  -11-18 yrs  -Inpt, outpt, cmty  -MDD  -Healthy controls, Psychiatric controls | 61% | 62% | -Diagnostic interview, K-SADS  -Clinician rating scale or interview, CDRS | -Activation during social interaction task  -fMRI, Cyberball task | -Not associated with inclusion-exclusion effects  -Associated across all interactions with blunted activity in insula, putamen, ACC, caudate, postcentral, precentral gyri  -Attempts associated across all interactions with greater activity in ACC, superior, middle frontal gyri | Fair |
| Oppenheimer et al. 2020 US [102] | Suicidality | Cross-sectional | -N = 36  -11-16 yrs  -Previous study  -Any anxiety disorder | 53% | 93% | Self-report instrument, MFQ | -Activation during social interaction  -fMRI, Simulated online chat room | -Ideation not associated with main effect in rejection  -Ideation associated greater activation anterior insula only if higher peer victimization or daily negative social experiences | Fair |
| Plener et al. 2012 Germany [103] | NSSI | Case-Control | -N = 18  -14-18 yrs  -Outpt, cmty  -None  -Healthy controls | 100% | Not given | Self-report instrument, FASM, OSI, SHBG | -Activation during emotion processing  -fMRI, IAPS (+ NSSI photos) | -Associated with greater activation in amygdala, hippocampus, bilateral ACC with IAPS emotional photos  -Explained by comorbid MDD  -Associated with less activation mOFC, IFC, MFC to IAPS emotional photos  -Associated greater activation mOFC, IFC, MFC with NSSI photos | Fair |
| Groschwitz et al. 2016 Germany [104] | NSSI | Case-Control | -N = 43  -11-18 yrs  -Inpt, outpt  -MDD  -Healthy controls, Psychiatric controls | 79% | Not given | Clinician rating scale or interview, SITBI | -Activation during social interactions  -fMRI, Cyberball task | -Associated in exclusion with increased activation mPFC, vlPFC, parahippo-campus | Fair |
| Quevdo et al. 2016b US [105] | NSSI | Case-Control | -N = 123  -11-18 yrs  -Inpt, outpt, cmty  -MDD  -Healthy controls, Psychiatric controls | 54% | Not given | -Diagnostic interview, K-SADS  -Clinician rating scale or interview, CDRS | -Activation during self-identity task  -fMRI, ISPT | -Associated for all perspectives with greater activation limbic, CMS  -Associated for mother’s perspective with greater activation limbic structures  -Associated for peers’ perspective with greater activation PCC,  precuneus | Fair |
| Brown et al. 2017 Germany [106] | NSSI | Case-Control | -N = 43  -11-28 yrs (separate adolescent data, 11-19 yrs)  -Inpt, outpt  -MDD  -Healthy controls | 89% | Not given | Clinician rating scale or interview, SITBI | -Activation during social interactions  -fMRI, Cyberball task | -Associated with greater activation of putamen in exclusion vs. inclusion, exclusion vs. passive viewing compared to healthy controls | Fair |
| Perini et al. 2019 Sweden [107] | NSSI | Case-Control | -N = 60  -15-18 yrs  - Outpt, cmty  -None  -Healthy controls | 100% | Not given | Clinician rating scale or interview, CANDI | -Activation during social interactions  -fMRI, Simulated online social interaction | -dmPFC, PCC, sgACC function during social anticipation predicted group membership | Fair |
| Poon et al. 2018 [108] | NSSI | Cross-sectional study | -N = 71  -12-14 yrs  -Cmty  -None | 52.5% | 75% | Clinician rating scale or interview, SITBI | -Activation during reward processing  -fMRI, Card-guessing task | -Associated with greater activation left, right putamen reward condition | Fair |
| Sauder et al., 2016 US [109] | Any self-harm | Case-Control | -N = 38  -13-19 yrs  -Previous studies, outpt, cmty  -None  -Healthy controls | 100% | 63% | Self-report instrument, SIQ  Clinician rating scale or interview, L-SASI | -Activation during response to reward  -fMRI, MID Task | -Associated with decreased activation in putamen, OFC, bilateral amygdalae during reward anticipation | Fair |
| *Functional Connectivity* | | | | | | | | | |
| Pan et al. 2013a US [53] | Suicidality | Case-Control | -N = 44  -13-17 yrs  -Previous studies, registry, cmty  -MDD  -Healthy controls, Psychiatric controls | 57% | Not given | -Clinician rating scale or interview, CSSRS  -Self-report, SIQ, BSIS | -Functional connectivity facial emotion processing  -fMRI, FEEST | -Associated decreased connectivity from ACG to bilateral insulae during angry faces | Fair |
| Alarcon et al. 2019 US [110] | Suicidality | Case-Control | -N = 120  -11-18 yrs  -Inpt, outpt, cmty  -MDD  -Healthy controls, Psychiatric controls | 56% | 62% | -Diagnostic interview, K-SADS  -Clinician rating scale or interview, CDRS | -Functional connectivity during self-identity processing  -fMRI, ESOM-Q | -Suicidality associated greater connectivity between amygdala and dlPFC, dmPFC, precuneus  -Attempts associated greater connectivity left amygdala to rACC  -Ideation associated greater connectivity from right amygdala to rACC | Good |
| Ordaz et al. 2018 US [111] | Suicidality | Cross-sectional | -N = 40  -14-17 yrs  -Oupt, cmty  -MDD | 75% | 62% | Clinician rating scale or interview, CSSRS | -Intrinsic neural network coherence  -Resting sate fMRI | -Severity of lifetime suicidality associated with lower coherence ECN, DFN SN  -Adjusted analyses, ECN only | Fair |
| Schreiner al. 2018 US [112] | Suicidality | Cross-Sectional | -N = 58  -12-19 yrs  -Previous studies, outpt, cmty  -MDD | 79% | 71% | Self-report instrument, IDAS | -Resting-state functional connectivity  -fMRI, focus on precuneus, PCC | -Higher suicidality increased connectivity between left precuneus, left primary motor, somatosensory cortices, middle, superior frontal gyri.  -Higher suicidality decreased connectivity between left PCC, left cerebellum, LOC, temporal–occipital fusiform gyrus | Fair |
| Schwartz et al. 2019 US [113] | Suicidality | Cohort | -N = 33  -14-17 yrs  -Outpt, cmty  -MDD | 75% | 62% | Clinician rating scale or interview, CSSRS | -Change in intrinsic neural network coherence to predict suicidality  -Resting state fMRI | - Greater 6-mo improvement SN coherence predicted decrease in suicidality | Good |
| Santamarina-Perez et al. 2019 US [114] | NSSI | Cohort | - N = 40  - 12-17 yrs  - Outpt, cmty  -None  -Healthy controls (only used at baseline) | 85% | Not given | -Clinician rating scale or interview, CSSRS  -Self-report, SIQ | -Resting-state functional connectivity baseline  -baseline connectivity predict response to psychological intervention  -Resting state fMRI | -Associated reduced connectivity amygdala to medial PFC network baseline  -Greater negative connectivity, higher NSSI improvement post-therapy | Good |
| ***Brain Function, Non-Imaging*** | | | | | | | | | |
| *Event-related Potentials (ERPs)* | | | | | | | | | |
| Tavakoli et al. 2018 Canada [115] | Suicidality | Case-Control | -N = 24  -13-17 yrs  -Inpt, cmty  -None  -Healthy controls | 83% | Not given | Self-report instrument, SBQ-R | -Attention capture  -EEG, P3a ERP | -Associated lower threshold for involuntary attention switch | Fair |
| Tsypes, et al. 2019 US [116] | Suicidality | Case-Control | -N = 69  -7-11 yrs  -Cmty  -None  -Healthy controls | 49% | 68% | -Diagnostic interview, K-SADS  -Self-report instrument, CDI | -Reward responsiveness  -EEG, Guessing doors, RewP-ERP to rewards and losses | -Ideation associated with blunted RewP change in response to reward | Fair |
| Pegg et al. 2020 US [117] | Suicidality | Case-Control | - N = 58  -14-18 yrs  -Outpt  -MDD  -Psychiatric controls | 67% | 88% | Diagnostic interview, K-SADS | -Reward responsiveness  -EEG, Guessing doors, RewP ERP to rewards and losses | -Associated with enhanced RewP to rewards and to losses | Good |
| Tsypes et al. 2018 US [118] | NSSI | Case-Control | -N = 57  -7-11 yrs  -Cmty  -None  -Healthy controls | 40% | 58% | Diagnostic interview, K-SADS | -Reward responsiveness  -EEG, Guessing doors, FN ERP to rewards and losses | -Associated with more negative change to losses | Fair |
| *Brain Waves* | | | | | | | | | |
| Graae et al. 1996 US [119] | Suicidality | Case-Control | -N = 38  -12-17 yrs  -Outpt, cmty  -None  -Healthy controls | 100% | Not given, but all Hispanic | Self-report instrument,  PSIS, HASS | -Brain waves  -EEG, alpha symmetry | -Associated left > right posterior alpha asymmetry | Fair |
| *Intracortical Inhibition* | | | | | | | | | |
| Lewis et al. 2019 US [120] | Suicidality | Non-Controlled Pre-Post  Intervention | -N = 10  -13-17 yrs  -Previous study  -MDD | 60% | Not given | Clinician rating scale or interview, CSSRS | -Changes in brain GABA receptor_B_  -TMS testing, long-interval intracortical  inhibition | -Ideation decrease after antidepressants associated with increase GABA_B_ mediated cortical inhibition | Fair |
| ***Brain Structure*** | | | | | | | | | |
| *Gray Matter Volume* | | | | | | | | | |
| Ho et al. 2018  US [122] | Suicidality | Cohort | -N = 152  -9-13 yrs  -Cmty  -None | 59% | 51% | -Self-report instrument, SIQ  -Computer Task, IAT | - GMV caudate, NAcc, putamen  -MRI | -24-mo implicit suicidality associated with decreased GMV bilateral putamen, left caudate  -Not associated with explicit suicidality | Good |
| Ando et al. 2018 Germany [123] | NSSI | Case-Control | -N = 50  -14-18 yrs  -Inpt, outpt, cmty  -None  -Healthy controls | 100% | Not given | Clinician rating scale or interview, SITBI | -GMV frontolimbic regions  -MRI | -Associated with decreased GMV ACC and insula  -Not associated with any other regions | Fair |
| Beauchaine  et al. 2019  US [124] | Any self-harm | Case-Control | -N = 44  -13-19 yrs  -Outpt, cmty  -None  -Health controls | 100% | 64% | -Clinician rating scale or interview,  L-SASI  -Self-report, SIQ | -Cortical GMV whole brain analysis  -MRI | -Associated reduced GMV bilateral insular cortex, rIFG  -Not associated with differences total brain volume | Fair |
| *Gray and White Matter Volume* | | | | | | | | | |
| Pan et al, 2015 US [124] | Suicidality | Case-Control | -N = 100  -12-17 yrs  -Not given  -MDD  -Healthy controls,  Psychiatric controls | Not given | Not given | -Clinician rating scale or interview, CSSRS  -Self-report, SIQ, BSIS | -Cortical thickness, GMV, WMV  -MRI | -Associated reduced thickness rSTG  -Not associated with WMV differences | Fair |
| Goodman et al. 2011 US [125] | Any self-harm | Case-Control | -N = 26  -13-17 yrs  - Inpt, cmty  -BPD/MDD  -Healthy controls | 77% | Not given | Clinician rating scale or interview, L-SDS | -Reduced volume in BA24  -MRI | -Higher number attempts associated with smaller GMV + WMV BA24  -Higher number attempts associated with higher WMV BA23  -Not associated with GMV BA23 | Fair |
| *Pituitary Gland Volume* | | | | | | | | | |
| Jovev et al. 2008 US [126] | Any self-harm | Cross-sectional | -N = 20  -15-19 yrs  -Outpt  - BPD | 75% | Not given | Researchers’ interview | -HPA axis activation  -MRI, PGV | -Higher number of behaviors associated with greater PGV | Fair |

ACC - Anterior Cingulate Cortex, ACG - anterior cingulate gyrus, BA23 - Brodmann Area 23 (ventral posterior cingulate area), BA24 - Brodmann Area 24 (ventral anterior cingulate area), BPD - Borderline Personality Disorder, BSIS - Beck Suicide Intent Scale, CANDI - Clinician-Administered Non-Suicidal Self-Injury Disorder Index, CDI - Child Depression Inventory, CDRS - Child Depression Rating Scale, CMS - Cortical Midline Structures, Cmty – Community, CSSRS - Columbia Suicide Severity Rating Scale, DMN - Default Mode Network, dlPFC - Dorsolateral Prefrontal Cortex, dmPFC - Dorsomedial Prefrontal Cortex, ECN - Executive Control Network, EEG - Electroencephalogram, ERP - Event Related Potential, ESOM-Q - Emotion Self-Other Morph Query, FASM - Functional Assessment of Self-Mutilation, FEEST - Facial Expressions of Emotions Stimuli and Test, fMRI - Functional Magnetic Resonance Imaging, FN ERP - Feedback Negativity ERP, GABA - Gamma Aminobutyric Acid, GMV – Gray Matter volume, HASS - Harkavy Asnis Suicide Scale, IAPS - International Affective Picture System, IAT – Implicit AssociationTest, IDAS- Inventory of Depression and Anxiety Symptoms, IFC - Inferior Frontal Cortex, IGT - Iowa Gambling Task, Inpt - Inpatients, ISPT - Interpersonal Self Processing Task, K-SADS - Kiddie-Schedule for Affective Disorders and Schizophrenia, LOC - Left Occipital Cortex, L-SDS - Lifetime Self-Destructiveness Scale, L-SASI - Lifetime Suicide Attempt Self-Injury Interview, MDD - Major Depressive Disorder, MFC - medial frontal cortex, MFQ - Moods and Feelings Questionnaire, MID – Monetary Incentive Task, mOFC - Medial Orbitofrontal Cortex, MOG – medial occipital gyrus, mPFC - medial prefrontal cortex, MRI – Magnetic Resonance Imaging, Nacc – Nucleus Accumbens, NSSI - Non Suicidal Self-Injury, OFC – Orbitofrontal Cortex, OSI - Ottawa Self-Injury Inventory, Outpt – Outpatients, PGV - Pituitary Gland Volume, PCC - Posterior Cingulate Cortex, PF – Prefrontal, PSIS - Pierce Suicide Intent Scale, rACC - Rostral Anterior Cingulate Cortex, rdACG - Right Dorsal Anterior Cingulate Gyrus, RewP ERP - Reward Positivity ERP, rIFG - Right Inferior Frontal Gyrus, rMTG - Right Medial Temporal Gyrus, r-STG – Right Superior Temporal Gyrus, SBQ-R - Suicide Behaviors Questionnaire - Revised, sgACC - Subgenual Anterior Cingulate Cortex, SHBG - Self-Harm Behavior Questionnaire, SIQ - Suicidal Ideation Questionnaire, SITBI - Self-Injurious Thoughts and Behaviors Interview, SN - Salience Network, TMS - Transcranial Magnetic Stimulation, vlPFC - Ventrolateral Prefrontal Cortex, WMV - White Matter Volume, Yrs - Years
